# Parameters associated with unsuccessful pessary fitting for pelvic organ prolapse up to three months follow-up: a systematic review and meta-analysis

**DOI:** 10.1007/s00192-021-05015-2

**Published:** 2022-01-17

**Authors:** Claudia Manzini, Lisan M. Morsinkhof, C. Huub van der Vaart, Mariëlla I. J. Withagen, Anique T. M. Grob

**Affiliations:** 1 Department of Obstetrics and Gynecology, University Medical Centre Utrecht, Heidelberglaan 100, 3584 CX Utrecht, The Netherlands; 2 Utrecht, The Netherlands; 3 Magnetic Detection and Imaging, Faculty of Science and Technology, Technical Medical Centre, University of Twente, Enschede, The Netherlands; 4 Multi-Modality Medical Imaging, Faculty of Science and Technology, Technical Medical Centre, University of Twente, Enschede, The Netherlands

**Keywords:** Pelvic organ prolapse, Vaginal pessaries, Pessary fitting, Predictive factors, Predictive parameters, Patients’ characteristics

## Abstract

**Objectives:**

To clarify which parameters are associated with unsuccessful pessary fitting for pelvic organ prolapse (POP) at up to 3 months follow-up.

**Methods:**

Embase, PubMed and Cochrane CENTRAL library were searched in May 2020. Inclusion criteria were: (1) pessary fitting attempted in women with symptomatic POP; (2) pessary fitting success among the study outcomes with a maximal follow-up of 3 months; (3) baseline parameters compared between successful and unsuccessful group. A meta-analysis was performed using the random effects model.

**Main results:**

Twenty-four studies were included in the meta-analysis. Parameters associated with unsuccessful pessary fitting were: age (OR 0.70, 95% CI 0.56–0.86); BMI (OR 1.35, 95% CI 1.08–1.70); menopause (OR 0.65 95% CI 0.47–0.88); de novo stress urinary incontinence (OR 5.59, 95% CI 2.24–13.99); prior surgery, i.e. hysterectomy (OR 1.88, 95% CI 1.48–2.40), POP surgery (OR 2.13, 95% CI 1.34–3.38), pelvic surgery (OR 1.81, 05% CI 1.01–3.26) and incontinence surgery (OR 1.87, 95% CI 1.08–3.25); Colorectal-Anal Distress Inventory-8 scores (OR 1.92, 95% CI 1.22–3.02); solitary predominant posterior compartment POP (OR 1.59, 95% CI 1.08–2.35); total vaginal length (OR 0.56, 95% CI 0.32–0.97); wide introitus (OR 4.85, 95% CI 1.60–14.68); levator ani avulsion (OR 2.47, 95% CI 1.35–4.53) and hiatal area on maximum Valsalva (OR 1.89, 95% CI 1.27–2.80).

**Conclusion:**

During counselling for pessary treatment a higher risk of failure due to the aforementioned parameters should be discussed and modifiable parameters should be addressed. More research is needed on the association between anatomical parameters and specific reasons for unsuccessful pessary fitting.

## Introduction

Vaginal pessaries are widely used as a conservative treatment option in the management of pelvic organ prolapse (POP) [1, 2] and have proven effective in relieving POP symptoms [3–5]. However, multiple attempts with different pessaries are sometimes required before obtaining an adequate fit [6]. Additionally, pessary fitting is reported as unsuccessful in up to 59% of the women [7], the most common reasons being pessary dislodgment, discomfort/pain, de novo urinary symptoms and failure to relieve POP symptoms [8]. Many studies have been published on the factors associated with (un) successful pessary fitting for POP [7–39]. Among other potential predictors, age, body mass index (BMI), prior surgeries, predominant POP compartments and advanced POP have been assessed, but results differ across studies. It is thus necessary to clarify which parameters are associated with unsuccessful pessary fitting. This knowledge could improve the clinical practice of physicians dealing with POP: the counselling for pessary treatment would be more effective and more targeted, and potential parameters associated with failure would be known and discussed with the patient. In addition, modifiable factors could be addressed to increase the probability of success.

The aim of the current review and meta-analysis is to clarify which clinical, demographical and anatomical (assessed by clinical examination or imaging techniques) parameters are associated with unsuccessful pessary fitting for POP up to 3 months follow-up. A maximum of 3 months follow-up was chosen to focus on pessary fitting process instead of long-term pessary use.

## Methods

### Sources

The first author searched Emtree/MeSH terms and keywords related to prolapse, pessary and the exposures (i.e. parameters associated with unsuccessful pessary fitting) through Embase, PubMed and the Cochrane CENTRAL library. The outcome, e.g. unsuccessful pessary fitting, was not included in the search to avoid the risk of missing relevant records. The terms searched through Embase are reported in Table 1 (the same search strategy was translated to PubMed and Cochrane CENTRAL library). The final search was made on the 8 May 2020. No time restrictions were applied, while restrictions were used for language (i.e. English). All results were exported to RefWorks (Legacy version), and duplicates were removed. If an abstract and a paper reporting the same data were retrieved, the abstract was considered a duplicate and removed.

**Table 1:** Embase search strategy

Emtree terms	Prolapse	Pessary	Exposure(s)
	‘pelvic organ prolapse’ ‘pelvic floor prolapse’	‘vagina pessary’	parameters ‘prediction and forecasting’ ‘morphological trait’ ‘groups by age’ ‘body mass’ ‘body weight’ ‘gynecologic surgery’
Keywords	prolapse(s) cystocele ‘anterior vaginal wall prolapse’ ‘anterior compartment prolapse’ ‘uterus prolapse’ ‘uterine prolapse’ ‘descensus uteri’ ‘vault prolapse’ ‘apical prolapse’ ‘apical compartment prolapse’ rectocele enterocele ‘posterior vaginal wall prolapse’ ‘posterior compartment prolapse’	pessar*	predictor(s) factor(s) characteristic(s) parameter(s) age BMI weight surger(y,ies) hysterectom(y,ies) compartment(s) stage(s) TVL GH

BMI = body mass index; TVL = total vaginal length; GH = genital hiatus

### Eligibility criteria

Studies were included in which (1) pessary fitting was attempted in women with symptomatic POP (at least 80% of the study population had to have symptomatic POP), (2) one of the assessed outcomes was the success of “initial fitting” and/or “fitting process” with a maximal follow-up of 3 months (in the case of a longer follow-up, at least 80% of the unsuccessful group had to have discontinued the pessary within 3 months from the initial fitting) and (3) baseline parameters (i.e. clinical, demographic and anatomical parameters) were compared between the successful and unsuccessful group. Study design was not a selection criterion and studies reported only in conference abstracts were not excluded. In the following, “initial fitting” will refer to the first visit, which is considered successful if the patient leaves the clinic with a pessary that stays comfortably in place. “Fitting process” will refer to pessary use from initial fitting until a defined follow-up time. It is considered successful if the patient is still using the pessary at follow-up. “Pessary fitting” will refer to both initial fitting and fitting process, if no distinction between the two is needed.

### Study selection

To select records eligible for full text assessment, title and abstract were screened by the first and second author, independently from each other. Any disagreement was resolved by discussion and the opinion of a third party (last author). The full text of the selected records was independently assessed by the same two authors. Disagreements were again resolved by discussion and the opinion of a third party (last author). The authors of a record were contacted if the full text of their paper was not accessible either online or at our institutional library and if some relevant parts of the records were unclear [e.g. definition of pessary fitting (un)success, time to follow-up, statistical significance of the observed differences or incorrect numbers].

### Data extraction

A standardized data extraction form was created to retrieve the information relevant to the research question. The following data were extracted: reference (first author, year, journal citation), study design type, study setting, inclusion and exclusion criteria, sample size, prolapse assessment (i.e. Pelvic Organ Prolapse Quantification system or Baden-Walker), pessary types used, assessment of initial fitting and/or fitting process, definition of successful fitting, success rate, time to follow-up, parameters compared between successful and unsuccessful group, significant parameters on univariate analysis and significant parameters on multivariate analysis (if performed). In case a record reported follow-ups beyond 3 months, only the parameters relating to the follow-ups of the first 3 months were extracted.

### Assessment of risk of bias

The Newcastle-Ottawa Scale (NOS) for case-control studies was used to assess the risk of bias of the included full-text articles [40]. Records only available as abstracts (i.e. no full-text available) were not assessed because of the limited amount of information they can provide. The NOS is specifically designed for non-randomized studies. It consists of three domains: Selection, Comparability and Exposure. The maximum total score is nine (four for the Selection domain, two for the Comparability domain and three for the Exposure domain). The first item assessed in the Selection domain is the adequacy of case definition and requires an independent validation. Since the success of pessary fitting is mostly patient self-reported, and no independent validation is applicable, no points could be given to this item. Therefore, the maximum score for the Selection domain was 3. A standard criterion for what constitutes a high-quality study base on the NOS has not yet been established. Generally, a study scoring ≥ 7 is considered high quality [41]. However, since no studies could get the maximum score on the Selection domain, we used a score of ≥ 6 as definition of high-quality studies.

### Data synthesis

To produce a qualitative synthesis of the results, all parameters assessed on their association with unsuccessful pessary fitting were clustered in a limited number of domains. For each domain one table was produced enumerating all studies in which a specific parameter was assessed on univariate and/or multivariate analysis.

To assess pessary fitting success rate, the weighted success rate at different times to follow-up was calculated. Sub-analyses were made for those studies which excluded and included women with unsuccessful initial fitting.

A meta-analysis of the parameters compared between successful and unsuccessful group in at least two records was performed. All available studies were combined without making any distinction based on the time to follow-up. A study was not included in the meta-analysis if the necessary input data were not reported and if, after having contacted the authors, they did not provide the requested data. In case of overlap between study populations of two records, the record with the largest sample size reporting the parameter of interest was included in the analysis. The meta-analysis was done with the Comprehensive Meta-analysis (CMA) version 3 software. Input data for dichotomous variables were number of exposed (i.e. number of patients with a specific parameter, e.g. prior hysterectomy) and sample size of unsuccessful and successful group, when available, or odds ratio (OR) and confidence intervals. In the last case, unadjusted ORs were used in the meta-analysis. For continuous variable input data were mean, standard deviation (SD) and sample size of unsuccessful and successful group or, if a *t*-test was run to compare the two groups, *p* value and sample size of the two groups. If the data were reported as median and range (minimum-maximum) or interquartile range (IQR), the authors were contacted and asked for mean and SD. In case of no response, mean and SD would have to be imputed to include the study in the meta-analysis. At first, the meta-analysis was run excluding the studies that required data imputation. To test if the imputed data would have influenced the results, the meta-analysis was also run after data imputation. If the data were reported as median and range, the mean was imputed using the method described by Hozo et al. [42] and the SD was imputed using the method described by Wan et al. [43]. If the data were reported as median and IQR, mean and SD were derived using Wan’s method. Authors were also contacted if they reported a parameter as significant or not significant without providing quantitative data. A random effect model was applied for the analysis. The summary measure used was OR. Heterogeneity was assessed with Q test and I-squared. For the significant parameters the risk of publication bias was assessed with the trim and fill procedure [44]. The meta-analysis without data imputation is presented in the result section, while the meta-analysis with data imputation is reported in Appendix E.

The review was conducted in adherence to the PRISMA and MOOSE guidelines. The protocol of the review was not registered before implementation.

## Results

### Study selection

**Fig. 1:**
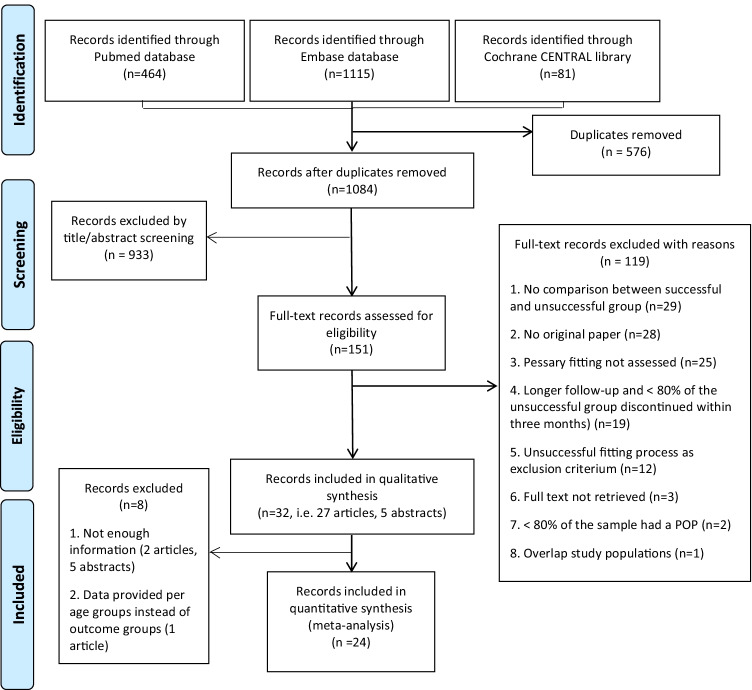
Records identification, inclusions and exclusions with reasons

Using the search strategy described, 1084 unique records were identified. The screening of title and abstract left 151 records. Of these, 119 were excluded after full text assessment and are reported in Appendix A. Thirty-two records (27 papers and five conference abstracts) were included in the qualitative synthesis and 24 in the meta-analysis (Fig. 1).

### Study characteristics

The characteristics of the 32 included records are enumerated in Table 2. In the following, the included records will be referred to according to the numbers reported in Table 2 and a superscript number will be used in the text. It has to be noted that there is an overlap between the study populations of Cheung et al. (2017) and Cheung et al. (2018) and Manchana (2011) and Manchana et al. (2012). In Appendix B the list of the authors contacted during the review process is reported.

**Table 2:** Characteristics of the included records

Journal papers: authors, year	Journal	Inclusion criteria	Exclusion criteria	N^*^	Pessary types	Study design	Set ting^*2^	Initial fitting/ fitting process	Definition of success	Follow-up^*3^		Success rate fitting	
										Study	Review	Initial	Process
(1) Cheung et al. 2017	UOG	- Symptomatic POP - No prior POP treatment - Double-ring pessary allowed - Maximum 3 re-fittings	- POP surgery or pessary removal within 1st year - No documented 1-year follow-up	255	Ring (double allowed)	Prospective observational	A	Fitting process	No pessary expulsion within 1 year (96% expulsion within 2 weeks)	1 year	2 weeks	–	59
(2) Cheung et al. 2018	Maturitas	- Symptomatic POP - No prior POP treatment - Double-ring pessary allowed - Maximum 3 re-fittings	- POP surgery or pessary removal within 1st year - No documented 1-year follow-up	528	Ring (double allowed)	Prospective observational	A	Fitting process	No pessary dislodgement within 1st year (94% dislodgment within 2 weeks)	1 year	2 weeks	–	69
(3) Clemons et al. 2004	AJOG	Symptomatic POP stage ≥ 2	–	100	Ring with diaphragm, Gellhorn, donut, double pessary	Prospective observational	A	Both combined	Pessary use 1 week after initial fitting/re-fitting (vs discontinuation within 2 weeks)	2 weeks		94	73
(4) Cundiff et al. 2007	AJOG	- Symptomatic POP stage ≥ 2 - Interest in non-surgical treatment	- Pregnancy - Prior pessary use - Vaginal narrowing or agglutination	134	Ring with support, Gellhorn	Randomized crossover trial	B	Both combined	Pessary use for 3 months	3 months		92	59 ^¤^
(5) Ding et al. 2015	IUJ	- Symptomatic POP stage 3–4 - Willingness to try a pessary	Unsuccessful initially fitting with a ring with support pessary	81	Ring with support	Prospective observational	C	Fitting process	Continued pessary use for > 3 months from the initial fitting	3 months		–	67
(6) Fernando et al. 2006	Obstet Gynecol	- Symptomatic POP - Willingness to try a pessary	- Willingness to undergo surgery - Non-English speaking, learning difficulties, dementia	203	Ring, cube, Gellhorn, donut	Prospective observational	A	Both combined	Reduction of POP without discomfort at the 2-week follow-up	2 weeks		–	75
(7) Geoffrion et al. 2013	Female Pelvic Med Reconstr Surg	Symptomatic POP	–	101	Ring with/without support (with/without knob), Gellhorn, oval, donut, Gehrung	Retrospective	A	Both combined	Pessary use after 4 weeks from initial fitting	4 weeks		78	74
(8) Jones et al. 2008	Obstet Gynecol	- Symptomatic POP - Willingness to non-surgical treatment	- Current pessary use - Pessary contraindications (active infection vagina or pelvis, undiagnosed vaginal bleeding, erosions, severe dementia)	90	Ring with support, Gellhorn, incontinence ring with knob, oval pessary	Prospective, observational, cohort	A	Both combined	Successfully continued pessary use at the 3-month visit	3 months		–	47
(9) Ko et al. 2011	J Minim Invas Gyn	- Symptomatic POP stage ≥ 2 - Successful initial fitting with a Gellhorn	Gynecological malignancy	46	Gellhorn	Retrospective	A	Fitting process	Pessary use for > 2 months	1 year	2 months	–	80
(10) Lekskulchai et al. 2015	J Med Assoc Thai	Women with POP treated with a pessary	Lost to follow-up before 3 months	194	Ring with/without support, donut, Gellhorn, pingpong ball	Retrospective chart review	A	Fitting process	Pessary use for > 3 months	3 months		–	84
(11) Maito et al. 2006	J Midwifery Womens Health	- POP and/or urinary incontinence (87% POP or both) - Willingness to try a pessary	–	120	Most common: ring with support	Retrospective chart review	E	Both combined	Comfortable pessary retained on Valsalva and void at the time of fitting/re-fitting (maximum 3 times)	17 months	Initial visit/refitting	90	86
(12) Manchana, 2011	Arch Gynecol Obstet	- Symptomatic POP - Willingness to try a pessary	–	100	Ring	Retrospective chart review	F	Both combined	Pessary use for > 2 weeks after initial fitting/re-fitting	13 months	2 weeks	77	62
(13) Manchana et al. 2012	IUJ	- Symptomatic POP - Willingness to try a pessary	–	126	Ring	Retrospective chart review	F	Both combined	Pessary use for > 2 weeks after initial fitting/re-fitting	1 year	2 weeks	–	61
(14) Mao et al. 2018	BJOG	- Symptomatic POP (stage ≥ 2) - Willingness to try a pessary (i.e. mainly contraindication/unwilling to undergo surgery, possible future pregnancy or > 60 years old)	–	343	Ring with support/ Gellhorn	Prospective observational	C	Both combined	Pessary use for > 2 weeks after initial fitting/re-fitting	2 weeks		92	88
(15) Markle et al. 2011	Female Pelvic Med Reconstr Surg	Symptomatic POP with/without urinary incontinence	Missing data	158	Gellhorn, Shaatz, incontinence dish or ring, ring (with/without support), cube, donut, Gehrung, Inflatoball, Regula, Smith-Hodge	Retrospective observational	C	Both combined	Pessary comfortably retained and plan to continue its use at 1-week follow-up	1 week		–	59
(16) Mokrzycki et al. 2001	J Low Genit Tract Di	- Symptomatic POP - Willingness to try a pessary	- Suspicion of gynaecological malignancy - Unexplained vaginal bleeding - Prior pessary use	42	Ring with support, cube, Gellhorn, Smith-Hodge, donut	Retrospective chart review	A	Fitting process	Ability and desire to continue pessary use at 3-month follow-up	3 months		–	57
(17) Mutone et al. 2005	AJOG	- Symptomatic POP - Trial of pessary management	Lost to follow-up (n = 23)	384	Ring with support, Gellhorn, cube, donut, Marland, Gehrung, Shaatz, Hodge, continence dish, regula, Inflatoball	Retrospective chart review	A	Both separate	1. Successful initial fitting 2. Patient still using the pessary at the 3 weeks follow-up and willing to continue	3 weeks		71	41
(18) Nemeth et al. 2013	IUJ	- Symptomatic POP stage ≥ 2 - Willingness to try a cube pessary as first-line treatment	- Undiagnosed vaginal bleeding - Vaginal erosions - Active vaginal infections - Dementia - Restricted mobility - Lost to follow-up (n = 6)	78	Cube	Prospective cohort	A	Fitting process	Pessary use at 1-year follow-up (vs discontinuation 2–4 weeks after initial visit)	1 year	2–4 weeks	97	71
(19) Nemeth et al. 2017	IUJ	- Symptomatic POP stage ≥ 2 - Women intended to be treated with a vaginal pessary	- Active infections of the pelvis or vagina - Inability to remove and reinsert the pessary - Unlikely to follow up	629	Cube, ring with/without support, ring with support and knob	Prospective cohort	A	Initial fitting	Successful initial fitting (vs failure to insert a pessary of appropriate size or loss/displacement during Valsalva)	Initial visit		96	–
(20) Nguyen et al. 2005	J WOCN	- Pelvic floor relaxation - Preference for nonsurgical management	–	130	Ring (with/without support), ring incont, Gellhorn, continence dish, Gehrung, cube, donut, regula	Retrospective chart review	C	Initial fitting	Successful initial fitting (vs inability to comfortably retain any pessary)	Initial visit		63	–
(21) Panman et al. 2017	IUJ	- Age ≥ 55 years - Symptomatic POP stage 2–3 - Women randomized to pessary (secondary analysis of a RCT)	- POP treatment in previous year - Current treatment for urogynecological disorders - Pelvic organ malignancy - Impaired mobility - Severe or terminal illness - Cognitive impairment - Insufficient Dutch language	78	Ring without/with support, Shaatz, Gellhorn	Cross-sectional	G	Both combined	Ability to wear the pessary for 2 weeks without any discomfort, regardless of the number of pessary trials	2 weeks		–	58
(22) Paterson et al. 2018	S Afr J Obstet Gynaecol	Symptomatic POP	–	73	Ring with support	Retrospective cross-sectional	A	Both combined	Pessary use for 6 month-s–1 year (vs ≤ 1 month)	1 year	1 month	–	–
(23) Ramsay et al. 2016	IUJ	- Symptomatic POP - ≥ 65 years, - Willingness to try a pessary	- Allergic to silicone - Unwilling to undergo conservative treatment - Incomplete medial record (n = 6)	304	Ring with support without/with knob, regula, donut, Shaatz, oval, Gehrung, Marland with support	Retrospective cohort	A	Both separate	1-Month pessary use with subjective improvement POP symptoms and no significant complications	12 years	1 month	–	63
(24) Turel et al. 2020	Aust N Z J Obstet Gynaecol	- Symptomatic POP - Willingness to try a pessary	- Obvious pessary contraindication - Incomplete dataset - Lost to follow-up	84	Ring	Retrospective	A	Both combined	Pessary still in situ without complications at 3-month follow-up	3 months	–	50	
(25) Wu et al. 1997	Obstet Gynecol	- Symptomatic POP - Willingness to try a pessary	–	110	Ring with/without support, cube	Prospective	C	Initial fitting	Successful initial fitting (i.e. pessary not expelled, patient could not feel the pessary, pessary did not descend to the introitus during testing)	4.5 years	Initial visit	74	–
(26) Yamada et al. 2011	J Obstet Gynaecol	- Uterine POP - Ring pessary treatment	–	69	Wallace ring pessary	Prospective	C	Fitting process	Pessary in situ for 4 weeks after the initial fitting (vs pessary expulsion)	1 month		–	77
(27) Yang et al. 2018	Arch Gynecol Obstet	Symptomatic POP	- Abnormal cervical cytology - Inflammation in the genital organs - Allergy to silicon	300	Ring with support, Gellhorn	Retrospective	F	Both combined	Retaining the pessary for 1 week without discomfort	8 years	1 week	–	83
Conference abstracts: authors, year	Journal	Inclusion criteria	Exclusion criteria	N*	Pessary types	Study design	Set ting*	Initial fitting/ fitting process	Definition of success	Follow-up^◊^		Success rate fitting	
										Study	Review	Initial	Process
(A) Cho et al. 2015	Female Pelvic Med Reconstr Surg	Pessary fitting for symptomatic POP	- Current pessary use without prior POPQ assessment - Pessary for SUI only - Prior pelvic radiation - Pregnant at pessary fitting - No documented 6-month follow-up	254	Support/space occupying	Retrospective cohort	A	Fitting process	Pessary continuation ≥ 4 weeks after initial fitting	4 weeks		**–**	65
(B) Hooper et al. 2018	Female Pelvic Med. Reconstr. Surg.	- Symptomatic POP - Successful initial fitting with a cube pessary	–	25	Cube	Prospective observational	D	Fitting process	Ability to retain the pessary for up to 1 week	1 week		**–**	No report
(C) Umachanger et al. 2018	IUJ	Symptomatic POP	–	130	Not specified	Retrospective chart review	C	Fitting process	Pessary use for > 3 months	3 months		**–**	67
(D) Triepels et al. 2019	Female Pelvic Med Reconstr Surg.	- POP stage ≥ 2 - Successful initial fitting	–	15	Not specified	Pilot	A	Fitting process	No pessary expulsion	< 3 months		**–**	–
(E) Zhu et al. 2011	IUJ	- Symptomatic POP - ring pessary	–	66	Ring without support	Prospective	C	Fitting process	Satisfactory pessary fitting	1 month and 3 months		**–**	73 and 65

*N = number of patients included in the analysis

*^2^ Setting = A: tertiary centre, B: multicentre, C: gynaecology department, D: urology department, E: nurse-midwifery pessary clinic, F: gynaecology clinic, G: general practice

*^3^ Follow-up: Study = longest time to follow-up assessed in the study; review = time to follow-up considered for the current review

¤ 59% = mean of the two trials of the randomized crossover trial

Abbreviations: POP = pelvic organ prolapse, SUI = stress urinary incontinence

### Risk of bias

In Table 3 the Newcastle-Ottawa Scale scores for the three domains and the total scores are reported. Mean total score was 6.

**Table 3:** Newcastle-Ottawa Scale scores

Papers	Selection maximum 4	Comparability maximum 2	Exposure maximum 3	Total score maximum 9
Cheung et al. 2017	2	2	3	7
Cheung et al. 2018	2	2	3	7
Clemons et al. 2004	3	0	3	6
Cundiff et al. 2007	3	0	3	6
Ding et al. 2015	3	0	3	6
Fernando et al. 2006	3	2	3	8
Geoffrion et al. 2013	2	2	2	6
Jones et al. 2008	3	2	3	8
Ko et al. 2011	2	0	2	4
Lekskulchai et al. 2015	3	0	2	5
Maito et al. 2006	3	2	2	7
Manchana, 2011	3	0	1	4
Manchana et al. 2012	3	0	1	4
Mao et al. 2018	3	2	3	8
Markle et al. 2011	3	1	2	7
Mokrzycki et al. 2001	2	0	2	4
Mutone et al. 2005	3	0	2	5
Nemeth et al. 2013	3	0	3	6
Nemeth et al. 2017	3	2	3	8
Nguyen et al. 2005	3	1	2	6
Panman et al. 2017	3	2	3	8
Paterson et al. 2018	2	0	2	4
Ramsay et al. 2016	3	0	2	5
Wu et al. 1997	3	0	3	6
Yamada et al. 2011	3	0	3	6
Yang et al. 2018	3	0	2	5
Turel et al. 2020	3	2	2	7

### Synthesis of results: success rate

Pessary fitting success rate ranged from 41%^17^ to 96%^19^. In Table 4 the weighted means at different times to follow-up are shown. Sub-analyses were made for those studies which excluded and included women with unsuccessful initial fitting. When the unsuccessful initial fitting was included, the success rates were overall lower (data at 3–4 weeks and 3 months). No sub-analysis was run for studies assessing fitting process success rate at 1/2 weeks, because only one study excluded women with unsuccessful initial fitting^2^.

**Table 4:** Weighted mean of pessary fitting success rate at different times to follow-up. Study reference refers to Table 2

Time to follow-up	Success rate: weighted mean			Study reference
Initial fitting	86% (95% CI 78%–92%)			3, 4, 7, 11, 12, 14, 17–20, 25
1–2 weeks	72% (95% CI 64%–79%)			2, 3, 6, 13–15, 21, 27
3–4 weeks	65% (95% CI 53%–76%)	Unsuccessful initial fitting excluded	70% (95% CI 62%–76%)	18, 26, A
		Unsuccessful initial fitting included	60% (95% CI 40%–76%)	7, 17, 23
2 months	80% (95% CI 66%–89%)			9
3 months	63% (95% CI 53%–72%)	Unsuccessful initial fitting excluded	69% (95% CI 59%–78%)	5, 10, 16, C, E
		Unsuccessful initial fitting included	53% (95% CI 45%–66%)	4, 8, 24

### Synthesis of results: parameters

**Fig. 2:**
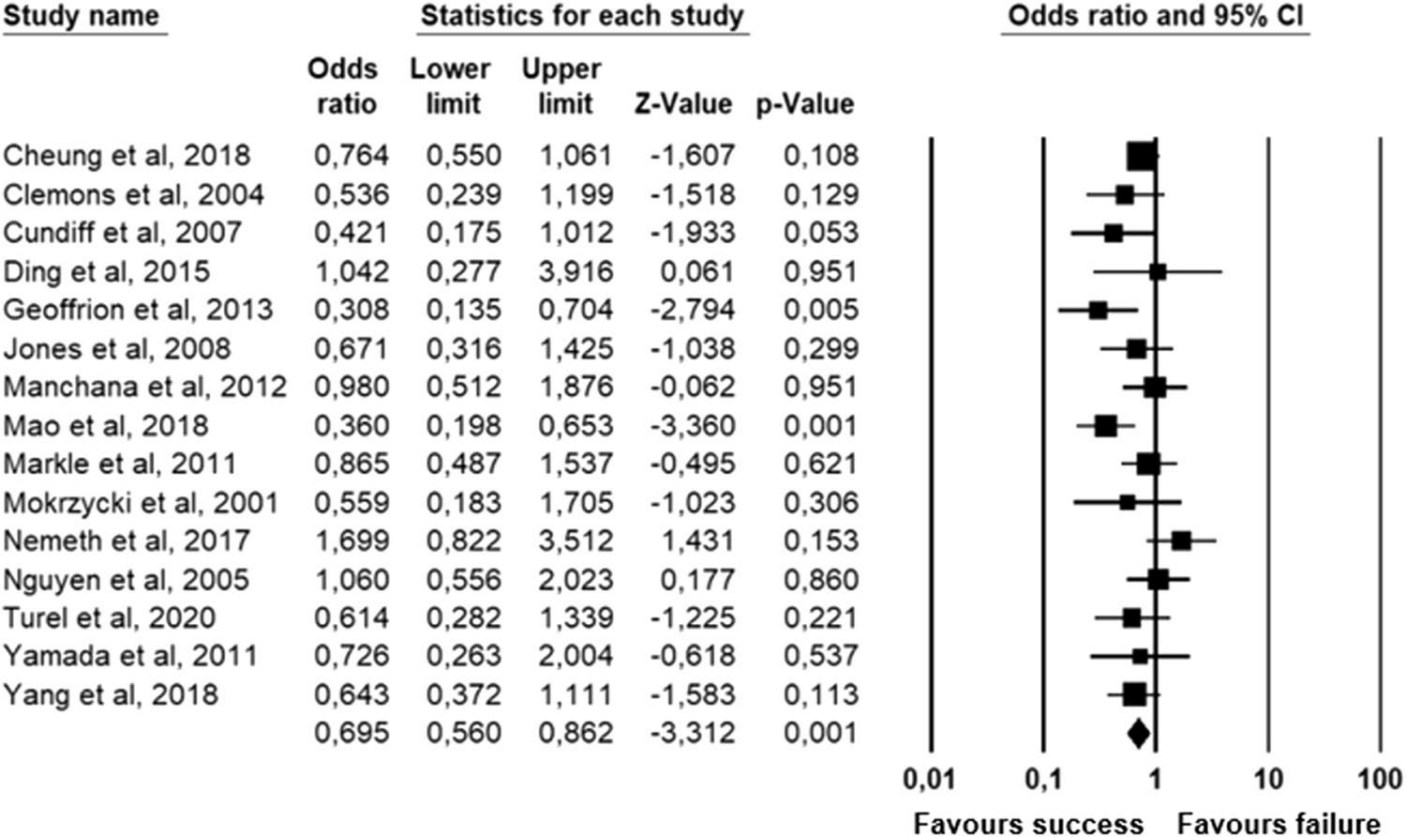
Forest plots of the significant parameters (results of the meta-analysis excluding imputed data)

**Fig. 3:**
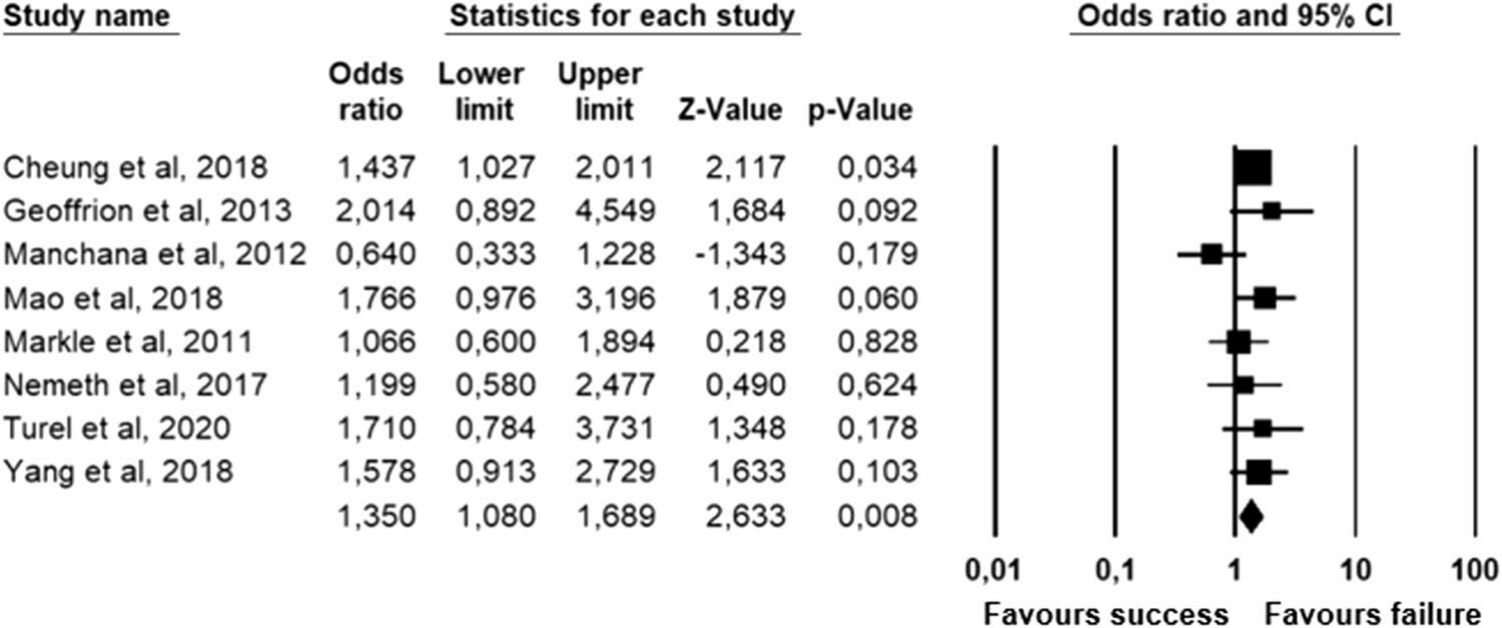
Forest plot for the association of **age** with successful pessary fitting up to 3-month follow-up (*N* = 2901)

**Fig. 4:**
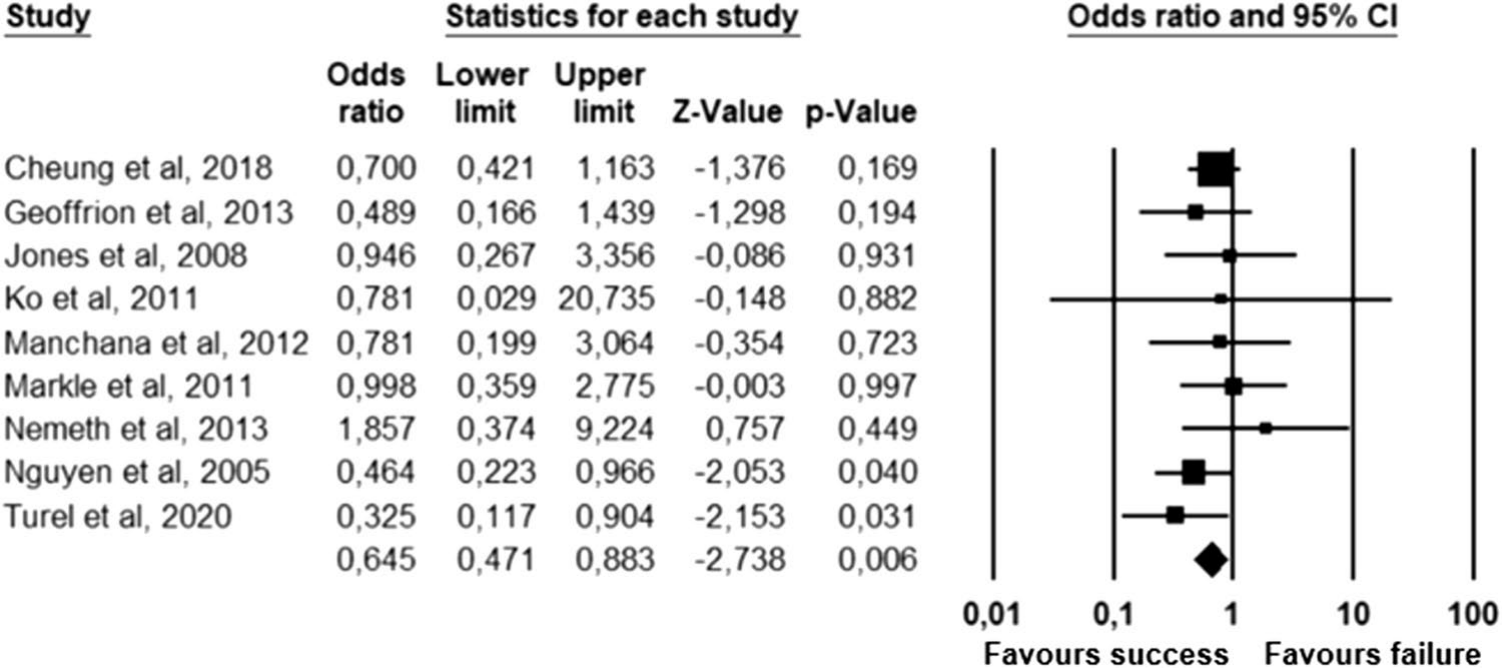
Forest plot for the association of **BMI** with unsuccessful pessary fitting up to 3-month follow-up (*N* = 2244)

**Fig. 5:**
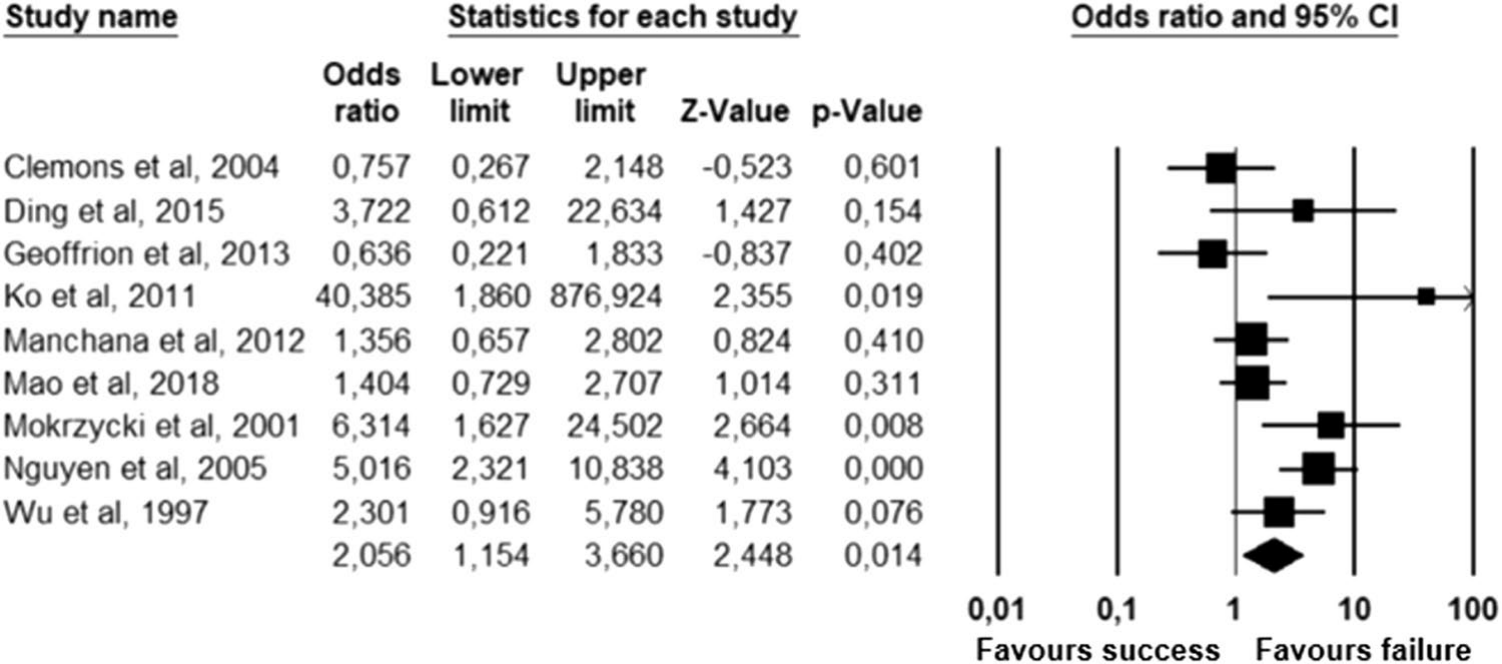
Forest plot for the association of **menopausal status** with successful pessary fitting up to 3-month follow-up (*N* = 1338)

**Fig. 6:**
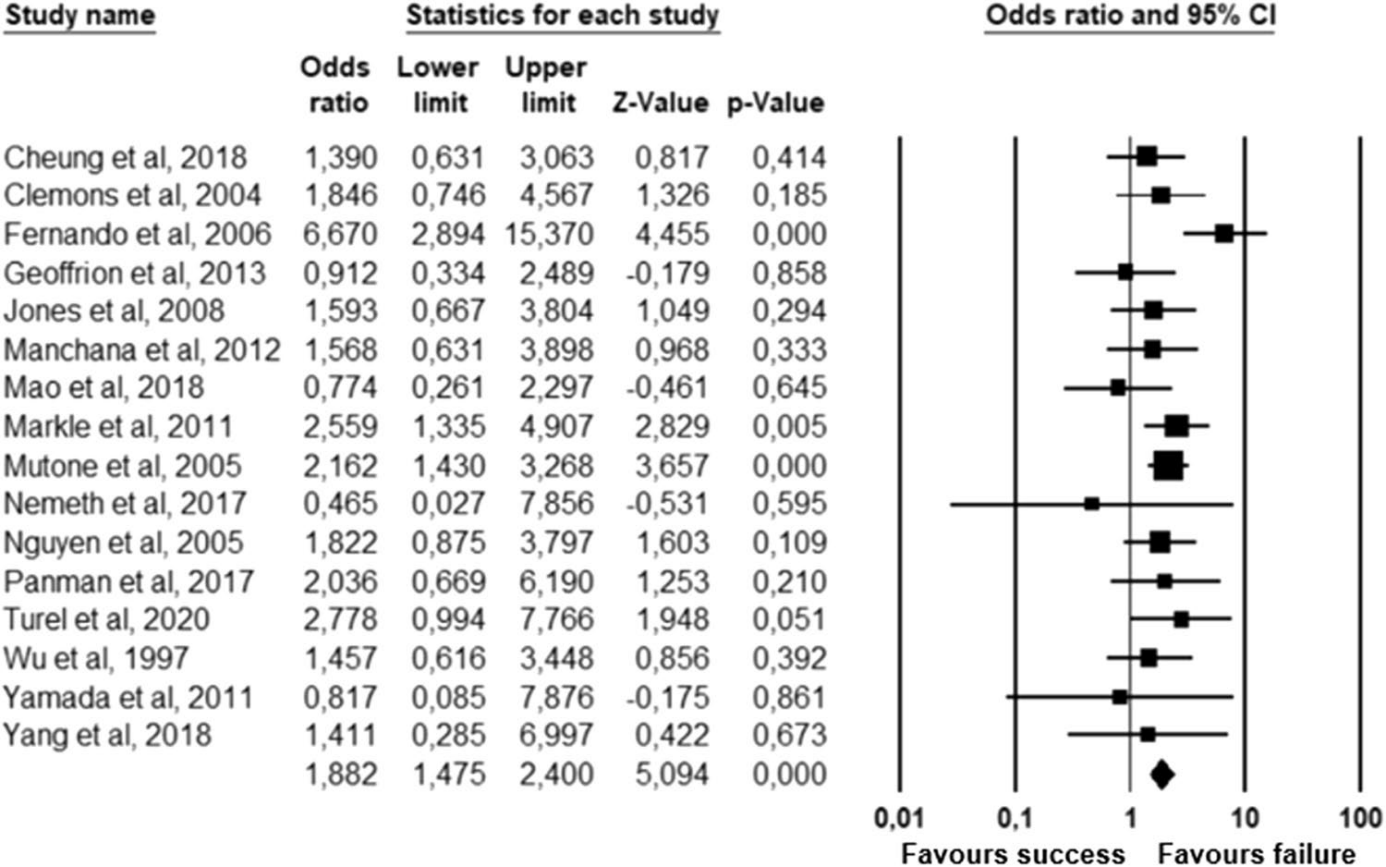
Forest plot for the association of **Stress urinary incontinence** (SUI) (i.e. pre-existing or de novo SUI) with unsuccessful pessary fitting up to 3-month follow-up (*N* = 1065)

**Fig. 7:**
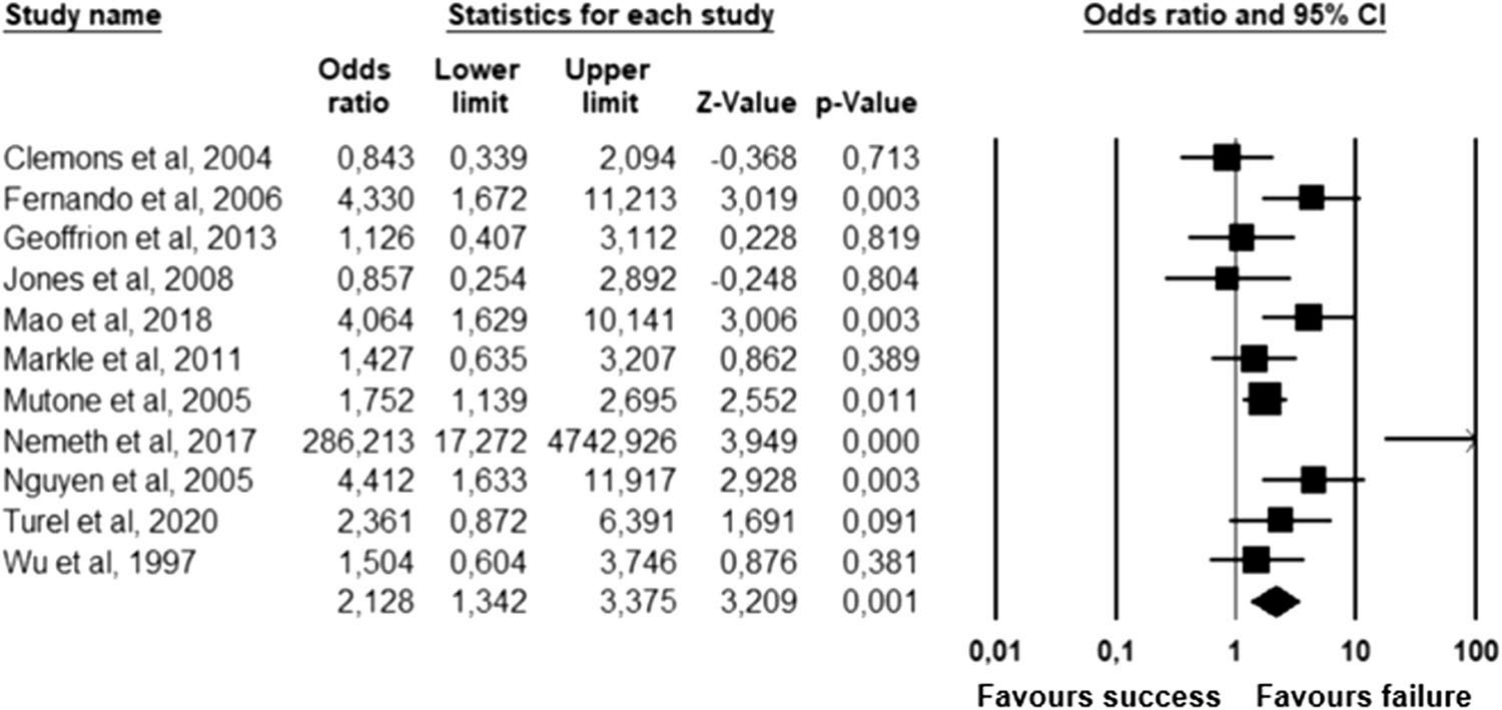
Forest plot for the association of **prior hysterectomy** with unsuccessful pessary fitting up to 3-month follow-up (*N* = 3431)

**Fig. 8:**
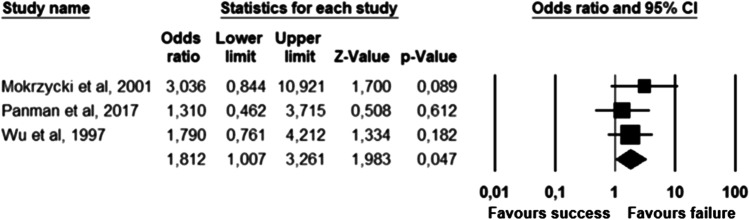
Forest plot for the association of **prior prolapse surgery** with unsuccessful pessary fitting up to 3-month follow-up (*N* = 2330)

**Fig. 9:**
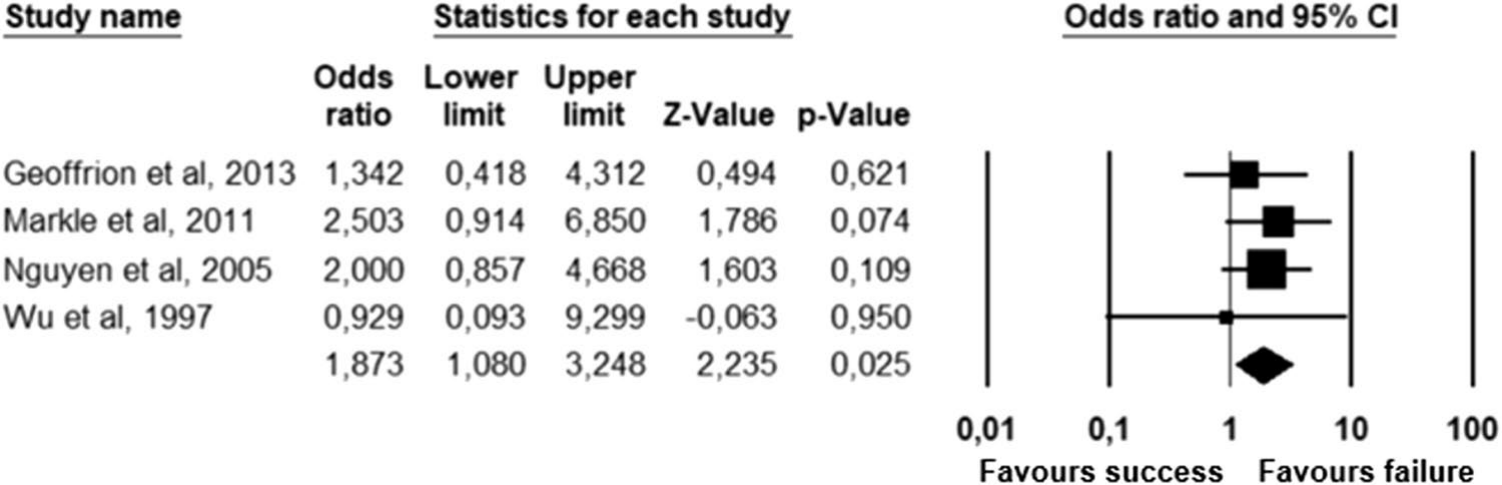
Forest plot for the association of **prior pelvic surgery** with unsuccessful pessary fitting up to 3-month follow-up (*N* = 230)

**Fig. 10:**
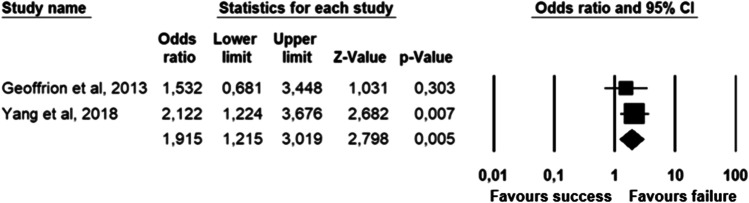
Forest plot for the association of **prior incontinence surgery** with unsuccessful pessary fitting up to 3-month follow-up (*N* = 497)

**Fig. 11:**
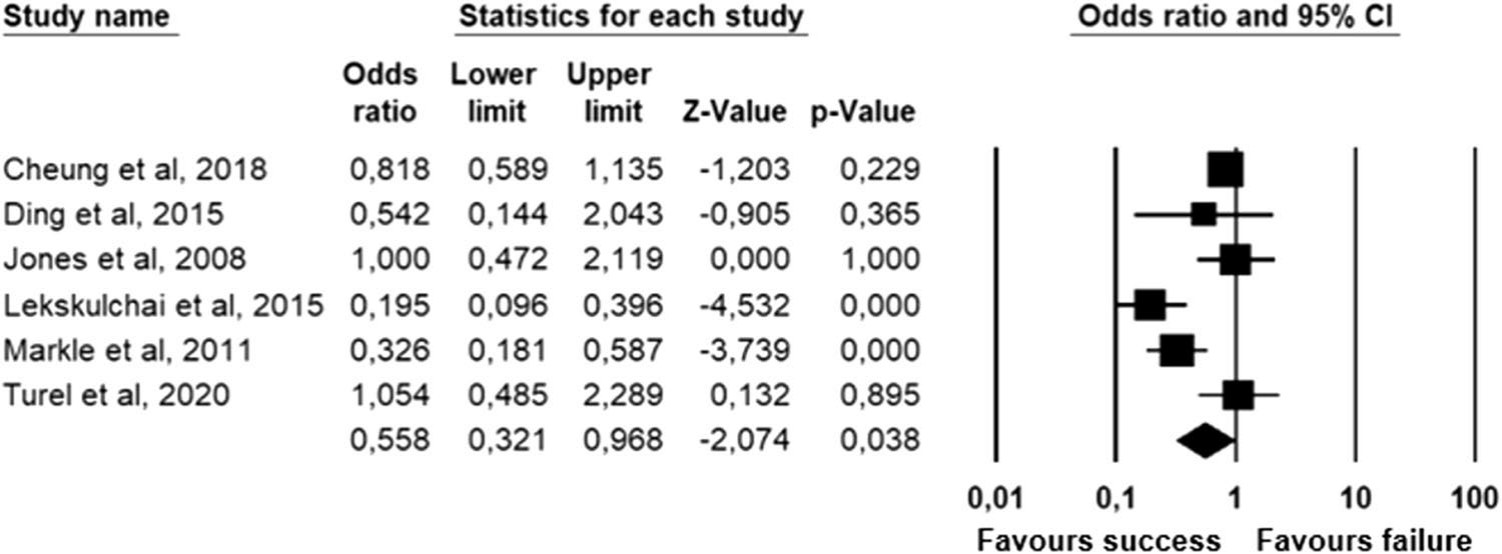
Forest plot for the association of **“CRADI-8”** (i.e. Colorectal-Anal Distress Inventory-8) scores with unsuccessful pessary fitting up to 3-month follow-up (*N* = 401)

**Fig. 12:**
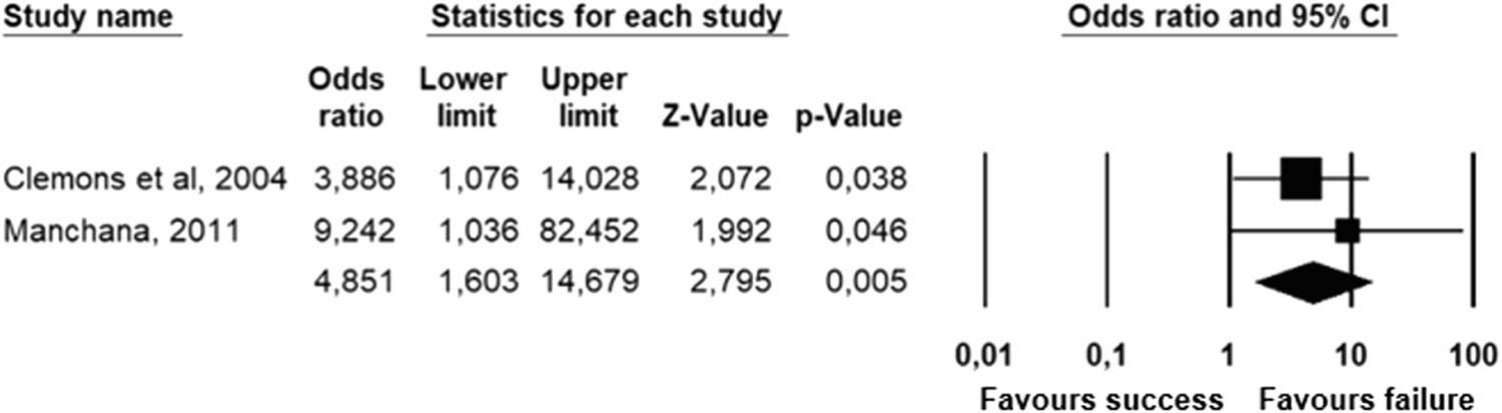
Forest plot for the association of **TVL** (i.e. total vaginal length) with successful pessary fitting up to 3-month follow-up (*N* = 1135)

**Fig. 13:**
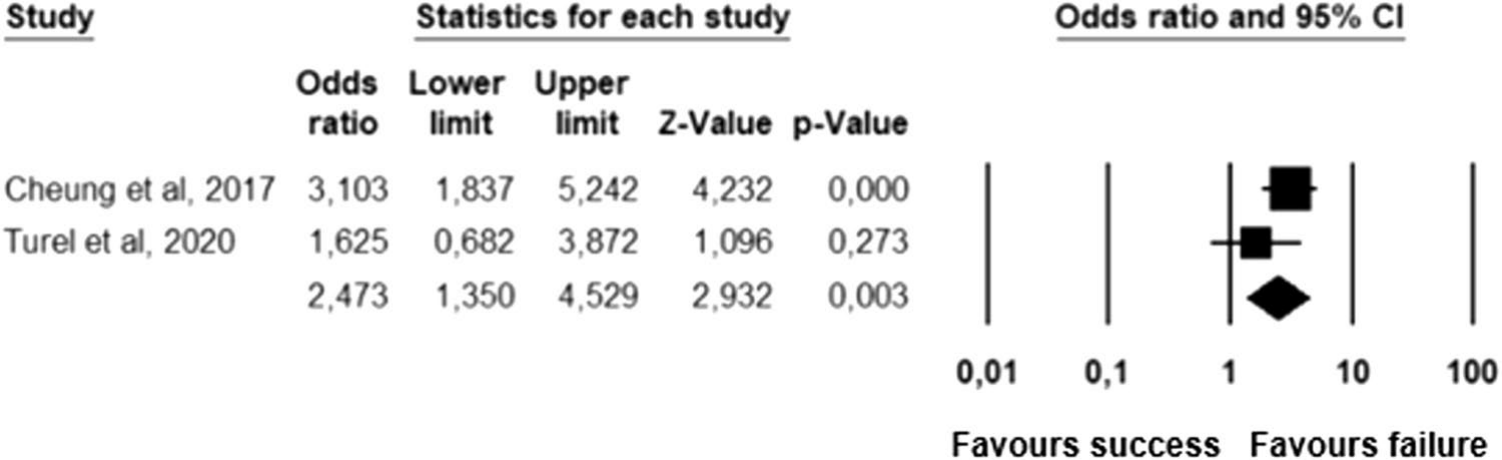
Forest plot for the association of **wide introitus** (i.e. ≥ 4 fingerbreadths) with unsuccessful pessary fitting up to 3-month follow-up (*N* = 200)

**Fig. 14:**
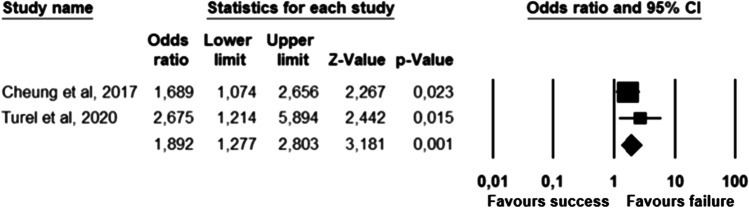
Forest plot for the association of **levator ani muscle avulsion** with unsuccessful pessary fitting up to 3-month follow-up (*N* = 339)

The parameters assessed on their association with unsuccessful pessary fitting by different authors were clustered into nine domains: (1) Demographics, (2) Obstetric history, (3) (Uro) gynaecological symptoms and medications, (4) Prior surgeries, (5) General history, (6) Questionnaires, (7) POP and pelvic floor assessment, (8) Pessary and (9) Imaging. Appendix C shows the domain tables enumerating all studies in which a specific parameter was assessed on univariate and/or multivariate analysis. The results of the meta-analysis excluding imputed data are shown in Table 5 and the corresponding forest plots in Figs. 2, 3, 4, 5, 6, 7, 8, 9, 10, 11, 12, 13, 14 (significant parameters) and Appendix D (non-significant parameters).

**Table 5:** Results of the meta-analysis (imputed data excluded)

Parameter	OR (95% CI)	z-value	p value	Heterogeneity				Trim and fill		Study number
				Q value	df (Q)	p value	I-squared	OR (95% CI)	Q value	
**Demographics**										
**Age**	0.70 (0.56–0.86)	−3.31	**0.00**	20.14	14	0.13	30.49	0.70 (0.56–0.86)	20.14	2, 3, 4, 5, 7, 8, 13, 14, 15, 16, 19, 20, 24, 26, 27
**BMI**	1.35 (1.08–1.70)	2.63	**0.01**	8.30	7	0.31	15.70	1.31 (1.05–1.63)	9.49	2, 7, 13, 14, 15, 19, 24, 27
**Menopause**	0.65 (0.47–0.88)	−2.74	**0.01**	5.66	8	0.69	0.00	0.65 (0.47–0.88)	5.66	2, 7, 8, 9, 13, 15, 18, 20, 24
White ethnicity	0.96 (0.29–3.23)	−0.07	0.95	10.19	3	0.02	70.56	–	–	3, 4, 6, 7
**Obstetric history**										
No. pregnancies	0.71 (0.45–1.12)	−1.48	0.14	0.02	1	0.89	0.00	–	–	7, 27
No. deliveries	1.02 (0.62–1.67)	0.06	0.95	19.35	5	0.00	74.16	–	–	3, 6, 7, 19, 26, 27
No. vaginal deliveries	1.13 (0.73–1.74)	0.55	0.58	1.01	2	0.60	0.00	–	–	7, 15, 16
Largest baby°	1.65 (0.43–6.25)	0.73	0.46	6.99	2	0.03	71.39	–	–	5, 7, 14
**(Uro) gynaecological symptoms and medications**										
**Stress urinary incontinence**	2.06 (1.15–3.66)	2.45	**0.01**	22.33	8	0.00	64.18	1.88 (1.03–3.43)	26.28	3, 5, 7, 9, 13, 14, 16, 20, 14
Sexually active	1.27 (0.81–2.00)	1.04	0.30	9.46	5	0.09	47.17	–	–	2, 3, 7, 13, 15, 21
HRT	0.83 (0.51–1.35)	−0.75	0.45	9.25	5	0.10	45.94	–	–	3, 7, 8, 15, 20, 25
**Prior surgeries**										
**Prior hysterectomy**	1.88 (1.48–2.40)	5.09	**0.00**	17.99	15	0.26	16.63	1.88 (1.48–2.40)	17.99	2, 3, 6, 7, 8, 13, 14, 15, 17, 19, 20, 21, 24, 25, 26, 27
**Prior POP surgery**	2.13 (1.34–3.38)	3.21	**0.00**	27.30	10	0.00	63.37	2.13 (1.34–3.38)	27.30	3, 6, 7, 8, 14, 15, 17, 19, 20, 24, 25
**Prior pelvic surgery**	1.81 (1.01–3.26)	1.98	**0.05**	0.10	2	0.61	0.00	1.81 (1.01–3.26)	0.10	16, 21, 25
**Incontinence surgery**	1.87 (1.08–3.25)	2.24	**0.03**	1.01	3	0.80	0.00	1.87 (1.08–3.25)	1.01	7, 15, 20, 25
**General history**										
Smoking	1.65 (0.97–2.81)	1.85	0.64	3.16	4	0.53	0.00	–	–	5, 7, 20, 21, 24
**Questionnaires**										
**CRADI-8**	1.92 (1.22–3.02)	2.80	**0.01**	0.42	1	0.52	0.00	nm	nm	7, 27
**POP and pelvic floor assessment**										
Predominant anterior compartment POP*	0.69 (0.40–1.19)	−1.34	0.19	24.21	7	0.00	71.09	–	–	2, 5, 8, 14, 16, 17, 21, 26
Predominant apical compartment POP*	1.31 (0.60–2.15)	0.38	0.71	16.14	5	0.01	69.02	–	–	2, 5, 8, 14, 17, 21
Predominant posterior compartment POP*	1.78 (0.98–3.24)	1.88	0.06	13.85	6	0.03	56.68	–	–	2, 8, 14, 16, 17, 21, 26
POPQ stadium 3–4	1.20 (0.62–2.31)	0.54	0.59	32.1	7	0.00	78.19	–	–	2, 3, 8, 9, 13, 14, 16, 17
**TVL**	0.56 (0.32–0.97)	−2.07	**0.04**	21.01	5	0.00	76.20	0.56 (0.32–0.97)	21.01	2, 5, 8, 10, 15, 24
GH	0.66 (1.25–2.39)	0.68	0.50	19.26	4	0.00	79.24	–	–	2, 5, 8, 15, 24
Perineal body	1.37 (0.83–2.28)	1.23	0.22	9.10	3	0.03	67.04	–	–	2, 8, 15, 24
Wide introitus**	4.85 (1.60–14.68)	2.80	**0.01**	0.45	1	0.50	0.00	nm	nm	3, 12
GH/TVL	1.87 (0.86–4.05)	1.58	0.12	4.86	2	0.09	58.85	–	–	5, 7, 15
Pelvic floor strength	0.88 (0.50–1.54)	−0.45	0.65	0.22	1	0.64	0.00	–	–	7,24
**Imaging**										
**Levator ani avulsion**	2.47 (1.35–4.53)	2.93	**0.00**	1.56	1	0.21	36.00	nm	nm	1, 24
**Hiatal area Valsalva**	1.89 (1.27–2.80)	3.18	**0.00**	0.98	1	0.32	0.00	nm	nm	1, 24

Bold = statistically significant; °largest baby > 8 lbs (studies 5, 7) or 4 kg (study 14). *In case of predominant multiple compartments (e.g. maximum POP stadium in the anterior and apical compartment), the patient was included in all relevant groups (e.g. predominant anterior compartment POP and predominant apical compartment POP). **Wide introitus ≥ 4 fingerbreadths; nm = not measurable (to run a publication bias procedure at least three studies must be included); HRT = hormone replacement therapy; POP = pelvic organ prolapse; CRADI-8 = Colorectal-Anal Distress Inventory-8. The study number refers to Table 2

Parameters associated with unsuccessful pessary fitting are: younger age, higher BMI, pre-menopausal status, stress urinary incontinence (SUI), prior surgery (i.e. hysterectomy, POP surgery, pelvic surgery, and incontinence surgery), higher Colorectal-Anal Distress Inventory-8 (CRADI-8) scores (which assess symptoms of obstructive defecation, anal incontinence, pain during defecation, faecal urgency and rectal bulging), shorter total vaginal length (TVL), wide introitus, levator ani avulsion and larger hiatal area on maximum Valsalva. The heterogeneity between studies and risk of publication bias is low for age, BMI, menopausal status, prior hysterectomy, prior pelvic surgery and prior incontinence surgery. SUI, prior POP surgery and TVL show a low risk of publication bias, but a relatively high heterogeneity between studies. For CRADI-8 scores, wide introitus, levator ani avulsion and hiatal area on Valsalva, the heterogeneity between studies is low, but the impact of publication bias could not be quantified because only two studies could be included in the analysis.

In Appendix E the results of the meta-analysis including imputed data are shown and in Appendix F the corresponding forest plots. Running the analysis without and with the imputed data did not qualitatively change the results: significant parameters remained significant and non-significant parameters remained non-significant. Sub-analyses were made for the parameters SUI and predominant posterior compartment. SUI is associated with unsuccessful pessary fitting (OR 2.06, 95% CI 1.15–3.66, z-value 2.45, *p* value 0.01). However, grouping the studies into those which assessed pre-existing SUI only and those which also assessed de novo SUI (alone or in combination with pre-existing SUI), de novo SUI remains significant (OR 5.59, 95% CI 2.24–13.99, z-value 3.68, *p* value 0.00), while pre-existing SUI does not (OR 1.44, 95% CI 0.88–2.36, z-value 1.45, *p* value 0.15) with small heterogeneity within groups (Q-value 11.17, *p* value 0.13).

Predominant posterior compartment is not associated with unsuccessful pessary fitting (OR 1.78, 95% CI 0.98–3.24, z-value 1.88, *p* value 0.06). However, in case of predominant multiple compartments (e.g. maximum POP stadium in the apical and posterior compartment), the patient was included in all relevant groups (e.g. predominant apical compartment POP and predominant posterior compartment POP). Analysing solitary predominant posterior compartment POP (i.e. excluding women with multiple predominant compartments), a significant association with unsuccessful fitting is observed (OR 1.59, 95% CI 1.08–2.35, z-value 2.37, *p* value 0.02, Q-value 4.51, df (Q) 5, Q-test *p* value 0.48, I-squared 0.00) with low risk of publication bias (trim and fill procedure: OR 1.75, 95% CI 1.21–2.53, Q-value 7.04).

## Discussion

The aim of the current review and meta-analysis was to clarify which clinical, demographical and anatomical parameters are associated with unsuccessful pessary fitting for POP up to 3 months follow-up.

### Main findings: success rate

In the current review the success rate of pessary fitting ranged from 41% to 96%. However, these differences become smaller if sub-analyses are made based on the follow-up time. From initial fitting to 3 to 4 weeks follow-up, the mean success rate decreased from 86% (95% CI 78%–92%) to 65% (95% CI 54%–75%). Interestingly, after 4 weeks the success rate remained substantially stable [success rate of 63% (95% CI 53%–72%) at 3 months follow-up]. This suggests that planning a follow-up at 4 weeks after initial fitting would ensure the vast majority of the unsuccessful fittings were identified (as also reported by Lone et al. [45]). Studies in which only women with successful initial fitting were included reported higher success rates compared to studies in which also women with unsuccessful initial fitting were included. Therefore, our suggestion for future research is to clearly report whether this selection is made or not.

### Main findings: parameters

Parameters associated with unsuccessful pessary fitting include: younger age, higher BMI, pre-menopausal status, SUI, prior surgery (i.e. hysterectomy, POP surgery, pelvic surgery and incontinence surgery), higher CRADI-8 scores, shorter TVL, wide introitus, levator ani avulsion and larger hiatal area on maximum Valsalva.

In the case of SUI and prior POP surgery, the risk of publication bias is small, but the heterogeneity is relatively high. With respect to SUI, analysing separately the studies which assessed pre-existing SUI only, and those which also assessed de novo SUI, the heterogeneity within groups becomes smaller. Interestingly, de novo SUI remains significant, while pre-existing SUI does not. This suggests that pre-existing SUI alone is not associated with failure. Therefore, when counselling a patient for pessary treatment for POP, presence of pre-existing SUI should not be considered a reason for advising a different treatment. With respect to prior POP surgery, a possible explanation for the relatively high heterogeneity is that all women of the unsuccessful group in the study of Nemeth et al. (2017) had prior POP surgery with consequent extremely high OR in this study compared to the others.

Some parameters that are significant in the meta-analysis have to be taken with caution. First, TVL shows high heterogeneity between studies. Second, the impact of publication bias could not be quantified for CRADI-8, wide introitus, levator ani avulsion and hiatal area on Valsalva because only two studies could be included in the analysis. In addition, levator avulsion shows moderate heterogeneity, which can be explained by the different definitions of unsuccessful pessary fitting: pessary expulsion in the study of Cheung et al. and pessary discontinuation within 3 months follow-up in the study of Turel et al. The same explanation can be given to the moderate heterogeneity of other non-significant parameters, i.e. predominant apical compartment, advanced POP and GH. These parameters were associated with pessary dislodgment in the study of Cheung et al. but were not associated with unsuccessful pessary fitting when no distinction was made between different reasons for unsuccessful pessary fitting. The reasons for unsuccessful pessary fitting are numerous, e.g. dislodgment, discomfort/pain, de novo urinary symptoms and failure to relieve POP symptoms [8]. Some parameters could be associated only with specific reasons for pessary fitting failure, but not others; future research should analyse the association between anatomical parameters and individual causes of pessary fitting failure.

Parameters related to obstetric history, e.g. number of pregnancies, deliveries and vaginal deliveries, were not found to be associated with unsuccessful pessary fitting. However, no study assessed the influence of prior vaginal delivery vs no prior vaginal delivery on pessary fitting failure. If pessaries are supported by the pelvic floor muscles, prior vaginal delivery (which can cause pelvic floor muscles damage [46]) could be a risk factor for failure, even if POP mostly occurs in parous women. Being sexually active and hormone replacement therapy (HRT) use are not associated with (un) successful pessary fitting. Therefore, a sexually active woman with POP can be encouraged to try this treatment option and prescribing HRT only in case of indication is confirmed to be good practice.

Interestingly, advanced POP stage (3–4) is not associated with unsuccessful fitting. Therefore, pessary treatment can be advised to women with any stage of POP. Predominant anterior, apical or posterior compartment POPs are also not associated with unsuccessful fitting. However, higher CRADI-8 scores (which assess colorectal symptoms) and solitary predominant posterior compartment POP (i.e. maximum POP stage only in the posterior compartment, while women with multiple predominant compartments being excluded) are associated with unsuccessful fitting. These results confirm that pessary treatment is less effective in relieving colorectal symptoms [47].

Recently, a systematic review and meta-analysis has been published on the factors associated with unsuccessful pessary fitting in women with symptomatic POP [48]. Differences between their work and ours are the following. First, the follow-up for pessary fitting was 1 to 3 weeks in their work, while we included studies with a maximal follow-up of 3 months. Second, our search was performed in Embase, PubMed and Cochrane CENTRAL library, while theirs was performed in PubMed, and we screened 1084 records, while they screened 350. Third, they only included prospective studies, while we also included retrospective studies. Fourth, we assessed the weighted success rate of pessary fitting at different times to follow-up, which was not assessed in their work, while they assessed the reasons for pessary discontinuation after successful insertion, which we did not assess. Fifth, in our meta-analysis 24 studies were included, while 21 studies were included in theirs. Sixth, we performed a meta-analysis of 29 parameters, while they performed a meta-analysis of seven parameters. Seventh, we performed the analysis without and with data imputation, while they did not specify if imputed data were also included. With respect to the results, BMI and prior POP surgery were associated with pessary fitting failure in both works. In addition, GH was consistently not associated with pessary fitting failure. Different results were obtained for age, TVL, prior hysterectomy and advanced POP, which can be partially due to the differences described above. Furthermore, more studies were included in our meta-analysis, which should make our results more solid. Only three studies were included in the meta-analysis of the parameter “advanced POP” in their work. The one with the highest relative weight was the study of Cheung et al. in which the definition of failure was pessary dislodgment. It might be that advanced POP is a predictor of pessary dislodgment but not a predictor of other reasons for failure. Lastly, since we analysed more parameters, we also observed that menopausal status, de novo SUI, solitary predominant posterior compartment POP, higher CRADI-8 score, wide introitus, levator ani avulsion and larger hiatal area on maximum Valsalva are associated with unsuccessful pessary fitting.

### Strengths and limitations

The current review and meta-analysis has several strengths. It was conducted according to the PRISMA and MOOSE guidelines. Multiple databases were searched. Study selection was made, independently, by two authors. The included papers were, on average, high-quality studies with a low risk of bias, as assessed by the Newcastle-Ottawa Scale. Moreover, authors were contacted in the case of missing information. Some limitations have to be acknowledged. Meta-analyses have the limitation that the interaction between different parameters cannot be assessed. For example, it is highly probable that younger age and pre-menopausal status are correlated. However, we cannot establish whether one of the two is a confounder or both are independently associated with unsuccessful pessary fitting. In addition, mean and SD of continuous variables are needed to perform a meta-analysis, but some authors reported only median and range or median and IQR. To include these studies in the meta-analysis, mean and SD would have to be imputed. While we decided to exclude these studies from the meta-analysis to avoid any possible bias due to data imputation, we note that imputing mean and SD in these studies and including them do not qualitatively change the results: significant parameters remain significant and non-significant parameters remain non-significant. This suggests that our conclusions are robust.

### Conclusions

In women with symptomatic POP, younger age, higher BMI, pre-menopausal status, de novo SUI, prior surgery (i.e. hysterectomy, POP surgery, pelvic surgery or incontinence surgery), solitary predominant posterior compartment POP, presence of colorectal symptoms, shorter TVL, wide introitus, levator ani avulsion and larger hiatal area on maximum Valsalva are associated with unsuccessful pessary fitting up to 3 months follow-up.

These results do not imply that an alternative treatment should always be recommended to women with these characteristics, but rather that the higher risk of failure should be acknowledged and discussed during counselling for pessary treatment. Women with high risk of unsuccessful fitting because of, among others, a high BMI could work on this modifiable parameter to increase their probability of success, especially if they do not have many other treatment options (e.g. women who wish to have more children or those unwilling or not suitable to undergo surgery [49]). If pessary treatment is chosen, being aware of the higher risk of failure would relieve some of the frustration related to the unsuccessful pessary fitting process. One might object that such a counselling could lower women’s expectation thus increasing the risk of failure. However, any counselling should be evidence based and should allow women to make informed decisions to be ethical. In addition, the risk of pessary fitting failure should be weighted against the risks related to other treatments (e.g. surgery), which in many cases would encourage women to try pessary treatment.

Ethnicity, obstetric history, pre-existing SUI, sexual activity, use of HRT, smoking, predominant anterior, apical or multiple compartment POP, and advanced POP are not associated with unsuccessful pessary fitting. Therefore, women with these characteristics can be reassured that they do not have an increased risk of failure and can be encouraged to try pessary treatment.

With respect to the anatomical parameters (assessed by clinical examination or imaging techniques), more research is needed to investigate their association with specific reasons for unsuccessful pessary fitting, i.e. whether it is dislodgment, discomfort/pain or other reasons. In addition, only two studies included in the meta-analysis assessed the association between TPUS parameters and unsuccessful pessary fitting. Therefore, the added value of TPUS in the pessary fitting process should be further investigated.

## Appendix A List of the records excluded after full text assessment

1. Adams E, Thomson A, Maher C, Hagen S. Mechanical devices for pelvic organ prolapse in women. Cochrane Database Syst Rev. 2004:CD004010. 10.1002/14651858.CD004010.pub2.
2. Ai FF, Mao M, Zhang Y, Kang J, Zhu L. Effect of generalized anxiety disorders on the success of pessary treatment for pelvic organ prolapse. Int Urogynecol J 2018;29:1147–53. 10.1007/s00192-018-3562-1.
3. Ai F-F, Zhu L, Mao M, Zhang Y, Kang J. Depressive symptoms affect outcomes of pessary use in postmenopausal women with uterine prolapse. Climacteric 2018;21:184–8. 10.1080/13697137.2018.1430130.
4. Al-Badr A. Quality of life questionnaires for the assessment of pelvic organ prolapse: Use in clinical practice. LUTS Low Urin Tract Symptoms 2013;5:121–8. 10.1111/luts.12006.
5. Alnaif B, Drutz HP. The association of smoking with vaginal flora, urinary tract infection, pelvic floor prolapse, and post-void residual volumes. J Low Genit Tract Dis 2001;5:7–11. 10.1046/j.1526-0976.2001.51002.x.
6. Alperin M, Khan A, Dubina E, Tarnay C, Wu N, Pashos CL, et al. Patterns of pessary care and outcomes for medicare beneficiaries with pelvic organ prolapse. Female Pelvic Med Reconstr Surg 2013;19:142–7. 10.1097/SPV.0b013e31827e857c.
7. Anantawat T, Manonai J, Wattanayingcharoenchai R, Sarit-apirak S. Impact of a vaginal pessary on the quality of life in women with pelvic organ prolapse. Asian Biomed 2016;10:249–52. 10.5372/1905-7415.1003.487.
8. Armstrong K, Dessie S, Modest A, Hacker M, Hota L. The effect of vaginal oestrogen on pessary usage. Neurourol Urodyn 2014:18–21.
9. Atnip SD. Pessary use and management for pelvic organ prolapse. Obstet Gynecol Clin North Am 2009;36:541–63. 10.1016/j.ogc.2009.08.010.
10. Bai SW, Yoon BS, Kwon JY, Shin JS, Kim SK, Park KH. Survey of the characteristics and satisfaction degree of the patients using a pessary. Int Urogynecol J Pelvic Floor Dysfunct 2005;16:182–6; discussion 186. 10.1007/s00192-004-1226-9.
11. Bastani P, Solbian FE, Mokhtarkhani M, Yegani S, Kabiri N. Effects of vaginal pessaries on symptoms associated with pelvic organ prolapse. Int.J.Urol. 2014
12. Bastani P, Danandeh Osgui N. Improvement of concomitant symptoms of pelvic organ prolapsed with applied pessary. Clin Exp Obstet Gynecol 2019;46:42–4. 10.12891/ceog4309.2019.
13. Bezerra LS, Vasconcelos CT, Vasconcelos Neto J, Bezerra KD, Saboia DM, Oria MO, Do Nascimento TS, Reboucas RN,Viana VF. Adherence and follow-up to pessary using educational video: Prospective court. Int Urogynecol J 2017;28:S183–4.
14. Brincat C, Kenton K, Pat Fitzgerald M, Brubaker L. Sexual activity predicts continued pessary use. Am J Obstet Gynecol 2004;191:198–200. 10.1016/j.ajog.2004.03.083.
15. Broens-Oostveen MC, Mom RM, Lagro-Janssen ALM. [Genital prolapse; treatment and course in four general practices]. Ned Tijdschr Geneeskd 2004;148:1444–8.
16. Bugge C, Adams EJ, Gopinath D, Reid F. Pessaries (mechanical devices) for pelvic organ prolapse in women. Cochrane Database Syst Rev. 2013;2013. 10.1002/14651858.CD004010.pub3.
17. Bulchandani S, Toozs-Hobson P, Verghese T, Latthe P. Does vaginal oestrogen treatment with support pessaries in vaginal prolapse reduce complications? Post Reprod Heal 2015;21:141–5. 10.1177/2053369115614704.
18. Chan MC, Hyakutake M, Yaskina M, Schulz JA. What Are the Clinical Factors That Are Predictive of Persistent Pessary Use at 12 Months? J Obstet Gynaecol Can 2019;41:1276–81. 10.1016/j.jogc.2018.11.015.
19. Cheung R, Chan S, Lee L. Factors associated with dislodgment of vaginal pessary within one year. Int Urogynecol J Pelvic Floor Dysfunct 2016;27:S132–3.
20. Cheung R, Lee L, Chan S. Does levator ani muscle injury affect the efficacy of vaginal pessary in women with pelvic organ prolapse. Int Urogynecology J 281 Suppl 1 (S96-S97) Date Publ 1 Jun 2017 2018:20–2.
21. Cheung RY-K, Lee L-L, Chung TK-H, Chan SS-C. Predictors for dislodgment of vaginal pessary within one year in women with pelvic organ prolapse. Journal of Obstetrics and Gynaecology Research. 2017
22. Cheung RYK, Lee JHS, Lee LL, Chung TKH, Chan SSC. Vaginal Pessary in Women With Symptomatic Pelvic Organ Prolapse: A Randomized Controlled Trial. Obstet Gynecol 2016;128:73–80. 10.1097/AOG.0000000000001489.
23. Chien C-W, Lo T-S, Tseng L-H, Lin Y-H, Hsieh W-C, Lee S-J. Long-term Outcomes of Self-Management Gellhorn Pessary for Symptomatic Pelvic Organ Prolapse. Female Pelvic Med Reconstr Surg 2019. 10.1097/SPV.0000000000000770.
24. Clemons JL, Aguilar VC, Sokol ER, Jackson ND, Myers DL. Patient characteristics that are associated with continued pessary use versus surgery after 1 year. Am J Obstet Gynecol 2004;191:159–64. 10.1016/j.ajog.2004.04.048.
25. Coelho SCA, Marangoni-Junior M, Brito LGO, Castro EB de, Juliato CRT. Quality of life and vaginal symptoms of postmenopausal women using pessary for pelvic organ prolapse: a prospective study. Rev. Assoc Med Bras 2018;64:1103–7. 10.1590/1806-9282.64.12.1103.
26. Daneel L, te West NI, Moore KH. Does monthly self-removal of vaginal ring pessaries for stress incontinence/prolapse reduce complication rates? A 5 year audit. Neurourol Urodyn 2016:5–8.
27. Davila GW. Vaginal prolapse: management with nonsurgical techniques. Postgrad Med 1996;99:171–176,181,184–185.
28. Dekker JH, Burger H. Authors’ reply to the comment by Hanis et al. on “Predictors of unsuccessful pessary fitting in women with prolapse: a cross-sectional study in general practice,” by Panman et al. Int Urogynecol J 2017;28:1441. 10.1007/s00192-017-3421-5.
29. Deng M, Ding J, Ai F, Zhu L. Successful use of the Gellhorn pessary as a second-line pessary in women with advanced pelvic organ prolapse. Menopause 2017;24:1277–81. 10.1097/GME.0000000000000909.
30. Deng M, Ding J, Ai F, Zhu L. Clinical use of ring with support pessary for advanced pelvic organ prolapse and predictors of its short-term successful use. Menopause 2017;24:954–8. 10.1097/GME.0000000000000859.
31. Dengler EG, Mounsey LA, Gines F, Agha M, Long T, Geller EJ. Defecatory dysfunction and other clinical variables are predictors of pessary discontinuation. Int Urogynecol J 2019;30:1111–6. 10.1007/s00192-018-3777-1.
32. Dessie SG, Armstrong K, Modest AM, Hacker MR, Hota LS. Effect of vaginal oestrogen on pessary use. Int Urogynecol J 2016;27:1423–9. 10.1007/s00192-016-3000-1.
33. Ding J, Chen C, Song X-C, Zhang L, Deng M, Zhu L. Changes in Prolapse and Urinary Symptoms After Successful Fitting of a Ring Pessary With Support in Women With Advanced Pelvic Organ Prolapse: A Prospective Study. Urology 2016;87:70–5. 10.1016/j.urology.2015.07.025.
34. Ding J, Song X Chen, Deng M, Zhu L. Which factors should be considered in choosing pessary type and size for pelvic organ prolapse patients in a fitting trial? Int Urogynecol J 2016;27:1867–71. 10.1007/s00192-016-3051-3.
35. Duenas JL, Miceli A. Effectiveness of a continuous-use ring-shaped vaginal pessary without support for advanced pelvic organ prolapse in postmenopausal women. Int Urogynecol J 2018;29:1629–36. 10.1007/s00192-018-3586-6.
36. Dwyer L, Kearney R. Conservative management of pelvic organ prolapse. Obstet Gynaecol Reprod Med 2018;28:15–21. 10.1016/j.ogrm.2017.10.005.
37. Eberhard J, Geissbühler V. Pessary treatment in urogynaecology and obstetrics. Gynakol Prax 2002;26:119–33.
38. Espitia De La Hoz FJ. O-06 Evaluation of Quality of Life in Climacteric Women With Genital Prolapse after the Use of Pessary. J Sex Med 2017;14:e373–4. 10.1016/j.jsxm.2017.10.016.
39. Fitchett JR, Bhatta S, Sherpa TY, Malla BS, A Fitchett EJ, Samen A, et al. Non-surgical interventions for pelvic organ prolapse in rural Nepal: a prospective monitoring and evaluation study. JRSM Open 2015;6:2054270415608117. 10.1177/2054270415608117.
40. Friedman S, Sandhu KS, Wang C, Mikhail MS, Banks E. Factors influencing long-term pessary use: Reply by the authors. Int Urogynecol J 2011;22:1047. 10.1007/s00192-011-1393-4.
41. Friedman S, Sandhu KS, Wang C, Mikhail MS, Banks E. Factors influencing long-term pessary use. Int Urogynecol J 2010;21:673–8. 10.1007/s00192-009-1080-x.
42. Fritzinger KG, Newman DK, Dinkin E. Use of a pessary for the management of pelvic organ prolapse. Lippincotts Prim Care Pract 1997;1:431–6.
43. Geoffrion R, Zhang T, Lee T, Cundiff GW. Clinical characteristics associated with unsuccessful pessary fitting outcomes. Obstet Gynecol Surv 2014;69:79–81. 10.1097/01.ogx.0000444678.89257.5f.
44. Goodman R.W.;Komesu Y.M.;McFadden B.L.;Rogers R.G. Do pessaries prevent prolapse progression? Female Pelvic Med Reconstr Surg 182 SUPPL 1 (S25-S26) 2015:4–6.
45. Griebling TL. Re: Natural History of Pessary Use in Women Aged 65–74 versus 75 Years and Older with Pelvic Organ Prolapse: A 12-Year Study. J Urol 2017;197:1321. 10.1016/j.juro.2017.02.041.
46. Griebling TL. Vaginal pessaries for treatment of pelvic organ prolapse in elderly women. Curr Opin Urol 2016;26:201–6. 10.1097/MOU.0000000000000266.
47. Griffin M, Kim Y, Roberts R, Abed H. Pessary use as a first line treatment for pelvic floor disorders. Neurourol Urodynamics 34 SUPPL 1 (S74) 2015;34:S2–98. 10.1002/nau.22738.
48. Gupta P, Ehlert MJ, Bartley JM. Diagnosis and management of complex pelvic floor disorders in women. J Clin Outcomes Manag 2015;22:275–85.
49. Gupta A, Cox X, Dunivan GC, Gaskins JT, Rogers RG, Iglesia CB, et al. Desire for Continued Pessary Use Among Women of Hispanic and Non-Hispanic Ethnic Backgrounds for Pelvic Floor Disorders. Female Pelvic Med Reconstr Surg 2020;26:287–98. 10.1097/SPV.
50. Hagen S, Thakar R. Conservative management of pelvic organ prolapse. Obstet Gynaecol Reprod Med 2015;25:91–5. 10.1016/j.ogrm.2015.01.006.
51. Handa VL, Jones M. Do pessaries prevent the progression of pelvic organ prolapse? IUJ 2002;13:349–52. 10.1007/s001920200078.
52. Hanis SM, Khazaei S, Ayubi E, Mansori K. Comment on: Predictors of unsuccessful pessary fitting in women with prolapse: a cross-sectional study in general practice. Int Urogynecol J 2017;28:1439. 10.1007/s00192-017-3359-7.
53. Hanson L-AM, Schulz JA, Flood CG, Cooley B, Tam F. Vaginal pessaries in managing women with pelvic organ prolapse and urinary incontinence: patient characteristics and factors contributing to success. Int Urogynecol J Pelvic Floor Dysfunct 2006;17:155–9. 10.1007/s00192-005-1362-x.
54. Hassler A, Michael K. The pessary, a method for treating pelvic organ prolapse. J Diagnostic Med Sonogr 2005;21:12–6. 10.1177/8756479304272678.
55. Hooper GL. Person-Centered Care for Patients with Pessaries. Nurs Clin North Am 2018;53:289–301. 10.1016/j.cnur.2018.01.006.
56. Hsieh M-F, Tsai H-W, Liou W-S, Lo C-C, Lin Z-H, An Y-F, et al. Long-term compliance of vaginal pessaries: Does stress urinary incontinence matter? Medicine (Baltimore) 2019;98:e15063. 10.1097/MD.0000000000015063.
57. Jacobson NS, Taney J, Overbey J, Hardart A, J.A. F. Pessary use for managementofpelvic organ prolapse in atertiary care center: A retrospective review. Female Pelvic Med Reconstr Surg 235 Suppl 1(S90-S91)2017:3–5. 10.1097/SPV.0000000000000479.
58. Johannes O., Promberger R., Nmeth Z. Vaginal cube pessaries for genital prolapse. Climacteric 2014;17:48–108. 10.3109/13697137.2014.893733.
59. Jones KA, Harmanli O. Pessary use in pelvic organ prolapse and urinary incontinence. Rev. Obstet Gynecol 2010;3:3–9.
60. Komesu YM, Rogers RG, Rode MA, Craig EC, Gallegos KA, Montoya AR, et al. Pelvic floor symptom changes in pessary users. Am J Obstet Gynecol 2007;197:620.e1–620.e6. 10.1016/j.ajog.2007.08.013.
61. Komesu YM, Rogers RG, Rode MA, Craig EC, Schrader RM, Gallegos KA, et al. Patient-selected goal attainment for pessary wearers: what is the clinical relevance? Am J Obstet Gynecol 2008;198:577.e1–5. 10.1016/j.ajog.2007.12.033.
62. Krause H. Pessary use in pelvic organ prolapse. BJOG Int J Obstet Gynaecol. 2015
63. Kuhn A, Bapst D, Stadlmayr W, Vits K, Mueller MD. Sexual and organ function in patients with symptomatic prolapse: are pessaries helpful? Fertil Steril 2009;91:1914–8. 10.1016/j.fertnstert.2008.02.142.
64. Lalani S, Izett ML, Welford K, Lyons A, Kupelian A, Vashisht A. Outcomes of nurse-led pessary clinic for pelvic organ prolapse. Female Pelvic Med Reconstr Surg. 2019
65. Lamers BHC, Broekman BMW, Milani AL. Pessary treatment for pelvic organ prolapse and health-related quality of life: A review. Int Urogynecol J 2011;22:637–44. 10.1007/s00192-011-1390-7.
66. Lee HSJ, Cheung YR, Chan SS. Efficacy and complications of vaginal ring pessary in pelvic organ prolapse. Neurourol Urodyn 2014:1–2.
67. Lewthwaite BJ, Staley D, Girouard L, Maslow K. Characteristics of women with continued use of vaginal pessaries. Urol Nurs 2013;33:171–6.
68. Lone F, Thakar R, Sultan AH. One-year prospective comparison of vaginal pessaries and surgery for pelvic organ prolapse using the validated ICIQ-VS and ICIQ-UI (SF) questionnaires. Int Urogynecol J 2015;26:1305–12. 10.1007/s00192-015-2686-9.
69. Lone F, Thakar R, Sultan AH, Karamalis G. A 5-year prospective study of vaginal pessary use for pelvic organ prolapse. Int J Gynaecol Obstet 2011;114:56–9. 10.1016/j.ijgo.2011.02.006.
70. Lowenstein L, Gamble T, Sanses TVD, van Raalte H, Carberry C, Jakus S, et al. Changes in sexual function after treatment for prolapse are related to the improvement in body image perception. J Sex Med 2010;7:1023–8. 10.1111/j.1743-6109.2009.01586.x.
71. Manonai J, Sarit-Apirak S, Udomsubpayakul U. Vaginal ring pessary use for pelvic organ prolapse: Continuation rates and predictors of continued use. Menopause 2019;26:665–9. 10.1097/GME.0000000000001277.
72. Mao M, Ai F, Kang J, Zhang Y, Liang S, Zhou Y, et al. Successful long-term use of Gellhorn pessary and the effect on symptoms and quality of life in women with symptomatic pelvic organ prolapse. Menopause 2019;26:145–51. 10.1097/GME.0000000000001197.
73. Mao M, Ai F, Zhang Y, Kang J, Liang S, Xu T, et al. Changes in the symptoms and quality of life of women with symptomatic pelvic organ prolapse fitted with a ring with support pessary. Maturitas 2018;117:51–6. 10.1016/j.maturitas.2018.09.003.
74. Mao M, Xu T, Kang J, Zhang Y, Ai F, Zhou Y, et al. Factors associated with long-term pessary use in women with symptomatic pelvic organ prolapse. Climacteric 2019;22:478–82. 10.1080/13697137.2019.1582623.
75. Matsubara S. Factors influencing long-term pessary use: comment. Int Urogynecol J 2011;22:1045; author reply 1046. 10.1007/s00192-011-1394-3.
76. Nojima S, Wang L, S; Y Iwadare J, Nokazi N, Kanaeda T, et al. Long-term use of the vaginal ring pessary for pelvic organ prolapse-over 2 years follow up. Int Urogynecol J Pelvic Floor Dysfunct 2009;20 Suppl 3:241–491. 10.1007/s00192-009-0982-y.
77. Onwude JL. Genital prolapse in women. BMJ Clin Evid 2007;2007.
78. Onwude JL. Genital prolapse in women. BMJ Clin Evid 2009;2009.
79. Onwude JL. Genital prolapse in women. BMJ Clin Evid 2012;2012.
80. Panman CMCR, Wiegersma M, Kollen BJ, Berger MY, Lisman-van Leeuwen Y, Vermeulen KM, et al. Effectiveness and cost-effectiveness of pessary treatment compared with pelvic floor muscle training in older women with pelvic organ prolapse: 2-year follow-up of a randomized controlled trial in primary care. Menopause 2016;23:1307–18. 10.1097/GME.0000000000000706.
81. Patel M, Mellen C, O’Sullivan DM, LaSala CA. Impact of pessary use on prolapse symptoms, quality of life, and body image. Am J Obstet Gynecol 2010;202:499.e1–4. 10.1016/j.ajog.2010.01.019.
82. Patel MS, Mellen C, O’Sullivan DM, Lasala CA. Pessary use and impact on quality of life and body image. Female Pelvic Med Reconstr Surg 2011;17:298–301. 10.1097/SPV.0b013e31823a8186.
83. Pezzella M., Iervolino SA., Passaretta A., Torella M., Colacurci N. Sexual funstion among women using a pessary for pelvic organ prolapse. Neurourol Urodyn 2017;36:S5–87. 10.1002/nau.23302.
84. Pizarro-Berdichevsky J, Pattillo A, Blumel B, Gonzalez S, Arellano M, Cuevas R, et al. Prospective comparative results: Of pessary use in patients with symptomatic pelvic organ prolapse in patients above or below 65 years of age. Neurourol Urodyn 2015;34:S2–98. 10.1002/nau.22738.
85. Pizarro-Berdichevsky J, Pattillo A, Blumel B, Gonzalez S, Arellano M, Cuevas R, et al. Pessary use in patients with symptomatic pelvic organ prolapse-12 month prospective study of factors predictive of subjective improvement. Int Urogynecol J 2016;27:19–149. 10.1007/s00192-016-3042-4.
86. Poma PA. Nonsurgical management of genital prolapse. A review and recommendations for clinical practice. J Reprod Med 2000;45:789–97.
87. Powers K, Lazarou G, Wang A, LaCombe J, Bensinger G, Greston WM, et al. Pessary use in advanced pelvic organ prolapse. Int Urogynecol J Pelvic Floor Dysfunct 2006;17:160–4. 10.1007/s00192-005-1311-8.
88. Radnia N, Hajhashemi M, Eftekhar T, Deldar M, Mohajeri T, Sohbati S, et al. Patient Satisfaction and Symptoms Improvement in Women Using a Vginal Pessary for The Treatment of Pelvic Organ Prolapse. J Med Life 2019;12:271–5. 10.25122/jml-2019-0042.
89. Ralph C, Pohlhammer S, Blumel B, Pizarro-Berdichevsky J. Pessary use for symptomatic pelvic organ prolapse (SYMP POP) in patients <=65 years: Feasibility retrospective study. Neurourol Urodyn 2014;33:162–266. 10.1002/nau.22577.
90. Ramsay S, Bouchard F, Tu LM. Pelvic Organ Prolapse and Pessary Use: Is It a Viable Long Term Treatment Option? J Urol 2012;187:1–3. 10.1016/j.juro.2012.02.2310.
91. Ramsay S, Bouchard F, Tu LM, Sherbrooke U De. Long term outcomes of pessary use in women with pelvic organ prolapse. Neurourol Urodynamics 2011;30.
92. Robert M, Govan AJ, Lohani U, Uprety A. Feasibility of using pessaries for treatment of pelvic organ prolapse in rural Nepal. Int J Gynaecol Obstet 2017;136:325–30. 10.1002/ijgo.12061.
93. Robert M, Schulz JA, Harvey M-A. Technical update on pessary use. JOGC 2013;35:664–74. 10.1016/S1701-2163(15)30888-4.
94. Sarit-Apirak S, Manonai J. Vaginal pessary use for pelvic organ prolapse in thai women. Female Pelvic MedReconstrSurg 2014;20:151–368.
95. Sarma S, Ying T, Moore KH. Long-term vaginal ring pessary use: discontinuation rates and adverse events. BJOG 2009;116:1715–21. 10.1111/j.1471-0528.2009.02380.x.
96. Scarabelot K, Pereira F, Ghizzo L, Willing J, Virtuoso J. Use of pessary in the treatment of pelvic floor dysfunctions: A systematic review. Physiother Q 2018;26:1–8. 10.5114/pq.2018.74704.
97. Shayo BC, Masenga GG, Rasch V. Vaginal pessaries in the management of symptomatic pelvic organ prolapse in rural Kilimanjaro, Tanzania: a pre-post interventional study. Int Urogynecol J 2019;30:1313–21. 10.1007/s00192-018-3748-6.
98. Sitavarin S, Wattanayingcharoenchai R, Manonai J, Sarit-apirak S, Chittacharoen A. The characteristics and satisfaction of the patients using vaginal pessaries. J Med Assoc Thai 2009;92:744–7.
99. Srikrishna S, Cardozo L. How Best to Manage the Posterior Compartment. Curr. Bladder Dysfunct.Rep. 2013
100. Sulak PJ, Kuehl TJ, Shull BL. Vaginal pessaries and their use in pelvic relaxation. J Reprod Med 1993;38:919–23.
101. Takashima Y, Dancz C, Ozel B. Factors associated with pessary discontinuation: A retrospective review. Neurourol Urodyn 2016. 10.1002/nau.22967.
102. Tan GI, Balakrishnan S. Factors that predict the medium-term success of the use of pessaries for symptomatic pelvic organ prolapse. IntUrogynecolJ 2018;10:1–15.
103. Tasic L, Jurisic A, Jankovic S, Karadzov N. Vaginal pessary is the best option for some women. Maturitas 2017;100:182. 10.1016/j.maturitas.2017.03.213.
104. te West NID, Moore KH. Recent Developments in the Non-surgical Management of Pelvic Organ Prolapse. Curr Obstet Gynecol Rep 2014;3:172–9. 10.1007/s13669-014-0087-6.
105. Tso C, Lee W, Austin-Ketch T, Winkler H, Zitkus B. Nonsurgical Treatment Options for Women With Pelvic Organ Prolapse. Nurs Womens Health 2018;22:228–39. 10.1016/j.nwh.2018.03.007.
106. Urzua M, Alvarez J, Rondini C, M M. Pessary use in pelvic organ prolapse in women. Int Urogynecol J 2017;28:1–282. 10.1007/s00192-017-3337-0.
107. van Geelen JM, Dwyer PL. Where to for pelvic organ prolapse treatment after the FDA pronouncements? A systematic review of the recent literature. Int Urogynecol J 2013;24:707–18. 10.1007/s00192-012-2025-3.
108. Vanherpe P, Ghesquière S. Use of pessaries in the treatment of pelvic organ prolapse. Tijdschr Geneeskd 2015;71:596–603. 10.2143/TVG.71.09.2001854.
109. Williams KS, Wolff BJ, Lind LR, Winkler HA. Vaginal oestrogen reduces adverse events in patients with pelvic organ prolapse managed with a pessary. Female Pelvic Med Reconstr Surg 2015;21:S76–7.
110. Withayajiakkhajorn P, Limbutara W, Gorsagun C. Pessary expulsion rate and risk factors for expulsion in women with pelvic organ prolapsed in southern Thailand. Int Urogynecol J 2018;15:2017–9.
111. Wlazlak E, Kociszewski J, Krzycka M, Pazdrak M, Wlazlak W, Kluz T, et al. Daily used cube pessaries in women with pelvic organprolapse: Fitting, influence on pelvic floor function. Int Urogynecol J 2017;28:1–282. 10.1007/s00192-017-3337-0.
112. Wlazlak E, Kociszewski J, Krzycka M, Wlazlak W, Dunicz A, Surkont G. Ultrasound evaluation of the influence of cube pessaries on female’s pelvic floor. Neurourol Urodyn 2018:2018–21.
113. Wolff BJ., Williams KS., Lind LR., Winkler HA., Shalom DF. Predictive factors for long-term pessary use and discontinuation: A retrospective cohort study. Female Pelvic Med Reconstr Surg 2001;21:55–6.
114. Wolff B, Williams K, Winkler A, Lind L, Shalom D. Pessary types and discontinuation rates in patients with advanced pelvic organ prolapse. Int Urogynecol J 2017;28:993–7. 10.1007/s00192-016-3228-9.
115. Yimphong T, Temtanakitpaisan T, Buppasiri P, Chongsomchai C, Kanchaiyaphum S. Discontinuation rate and adverse events after 1 year of vaginal pessary use in women with pelvic organ prolapse. Int Urogynecol J 2018;29:1123–8. 10.1007/s00192-017-3445-x.
116. Zhu L, Mao M. Factors Associated with Long-Term Pessary Use in Women with Symptomatic Pelvic Organ Prolapse. J Minim Invasive Gynecol 2019;26:S210–1. 10.1016/j.jmig.2019.09.437.
117. Zhu L., Mao M., Ai F. Changes of symptoms and quality of life in women with symptomatic pelvic organ prolapse fitted with ring with support pessary: A long-term study. Int Urogynecol J 2018;10:1–15.
118. Li B, Chen Q, Zhang J, Yu C, Zhang L, Chen L. A prospective study of pessary use for severe pelvic organ prolapse: 3-year follow-up outcomes. Arch Gynecol Obstet 2020;301:1213–8. 10.1007/s00404-020-05526-1.
119. Yoshimura K, Morotomi N, Fukuda K, Kubo T, Taniguchi H. Changes of intravaginal microbiota and inflammation after self-replacement ring pessary therapy compared to continuous ring pessary usage for pelvic organ prolapse. J Obstet Gynaecol Res 2020;46:931–8. 10.1111/jog.14242.

## **Appendix B** List of the authors contacted during the review process

**Table:**

Phase	Authors	Reason contact	Response		Conclusion
Screening process	Triepels et al.	Unclear time to follow-up	Yes	< 3 months	Abstract included
	Yang et al.	Abstract on the same data reported in the paper?	Yes	Abstract on the same data reported in the paper	Abstract considered as a duplicate
	Eberhard et al.	Record not retrieved	No	–	Not included
	Fritzinger et al.	Record not retrieved	No	–	Not included
	Poma	Record not retrieved	No	–	Not included
Data analysis	Cheung et al.	Overlap study populations?^1,2^	Yes	Yes	Paper with the biggest sample for meta-analysis
	Cheung et al.	Missing data in the two groups on BMI and no. vaginal deliveries	Yes	BMI: 7 in drop pessary group and 18 in pessary group; vaginal delivery: 7 in drop pessary group and 27 in pessary group	Data used for meta-analysis
	Clemons et al.	Mean and SD of GH and TVL			Dichotomous data used in the analysis in which imputed data were included
	Cundiff et al.	Mean and SD of age, prevalence of white ethnicity in the two groups	Yes	Data provided	Data used for meta-analysis
	Ding et al.	Mean and SD of BMI, gravidity, parity	No		Data imputed for the analysis in which imputed data were included
	Fernando et al.	Mean and SD of age, parity	No		OR used as input data (age provided as dichotomous variable: only used in the analysis in which imputed data were included)
	Geoffrion et al.	Mean and SD of age	Yes	Data provided	Data used for meta-analysis
	Jones et al.	Mean and SD of parity	No		Data imputed for the analysis in which imputed data were included
	Ko et al.	Mean and SD of age, BMI, parity	No		Since data could not be imputed, not used for the analysis
	Lekskulchai et al.	Mean and SD of age, parity, length of the perineal body, GH	No		Data imputed for the analysis in which imputed data were included
	Maito et al.	Mean and SD of age, pelvic floor strength, vaginal parity; prevalence of prior POP procedure, hysterectomy, urinary incontinence procedure, grade 3 or 4 of POP, urinary incontinence in the two groups	No		Since data could not be imputed, study not included in the meta-analysis
	Manchana et al.	Overlap study populations?^11,12^	No	–	Paper with the biggest sample for meta-analysis
	Manchana et al.	Mean and SD of parity	No		Data imputed for the analysis in which imputed data were included
	Mao et al.	Mean and SD of gravidity, no. vaginal deliveries, TVL, vaginal introitus, GH; prevalence of menopausal women in the two groups	No		Data imputed for the analysis in which imputed data were included (when possible)
	Mokrzycki et al.	Mean and SD of vaginal parity	No		P value of a t-test and sample sizes of the two groups used as input data
	Mutone et al.	Mean and SD of age, BMI, levator ani strength, GH, perineal body length, TVL	Yes	Data not available	Data imputed and BMI used as dichotomous variable for the analysis in which imputed data were included
	Nemeth et al. 2013	Mean and SD of age, BMI, parity	No		Data imputed for the analysis in which imputed data were included (when possible)
	Nguyen et al.	SD of age, mean and SD of parity, number of women with pre-existing SUI and SUI after POP reduction in the two groups	No		P value of a t-test and sample sizes of the two groups used for age. Largest SD reported by other studies used for parity in the analysis in which imputed data were included. Groups with pre-existing SUI and SUI after POP could not be separated
	Panman et al.	Mean and SD of age, BMI, parity, CRADI-8, pelvic floor strength, GH, TVL	No		Data imputed for the analysis in which imputed data were included
	Paterson et al.	Mean and SD of hiatal distance on contraction. Data on levator avulsion.	No		Since data could not be imputed, study not included in the meta-analysis
	Ramsay et al.	Prevalence of prior hysterectomy, prior POP surgery, predominant anterior, apical, posterior POP compartment, POP stage 3–4, stress urinary incontinence in the two groups; mean and SD of number of vaginal deliveries	No		Since data could not be imputed, study not included in the meta-analysis
	Wu et al.	Mean and SD of age and parity	Yes	Data not available	Not included in the meta-analysis
	Cho et al.	Clarifications on their results	No	–	Results described according to our understanding. Conference abstract: not included in the meta-analysis
	Hooper et al.	Clarifications on their results	Yes	Grade of POP and prior hysterectomy predictors of unsuccessful fitting at 1 week	Results described according to authors. Conference abstract: not included in the meta-analysis

## Appendix C. Each table shows the parameters of one specific domain. For each parameter the studies in which it was assessed on univariate and/or multivariate analysis are reported as well as whether it was significant or not

### **Appendix C1** Demographics domain

**Table:**

Parameter	Univariate analysis assessment	Significant univariate analysis	Multivariate analysis Assessment	Significant multivariate analysis
Age	Cheung et al. 2017 Cheung et al. 2018 Clemons et al. Cundiff et al. Ding et al. Fernando et al. Geoffrion et al. Jones et al. Ko et al. Lekskulchai et al. Manchana, 2011 Manchana et al. 2012 Mao et al. Markle et al. Mokrzycki et al. Mutone et al. Nemeth et al. 2013 Nemeth et al. 2017 Nguyen et al. Ramsay et al. Wu et al. Yamada et al. Yang et al. Turel et al. Cho et al.	Cundiff et al. Fernando et al. Geoffrion et al. Mao et al. Wu et al.	Fernando et al. Geoffrion et al. Maito et al. Mao et al. Panman et al.	Geoffrion et al. Panman et al.
BMI/weight	Cheung et al. 2017 Cheung et al. 2018 Ding et al. Geoffrion et al. Ko et al. Lekskulchai et al. Manchana et al. 2012 Mao et al. Markle et al. Mutone et al. Nemeth et al. 2013 Nemeth et al. 2017 Nguyen et al. Yang et al. Turel et al. Cho et al.	Mao et al. Mutone et al.	Cheung et al. 2018 Maito et al. Mao et al. Panman et al.	Mao et al. Panman et al.
Menopause	Cheung et al. 2017 Cheung et al. 2018 Geoffrion et al. Jones et al. Ko et al. Manchana et al. 2012 Mao et al. Markle et al. Mokrzycki et al. Nemeth et al. 2013 Nguyen et al. Yang et al. Turel et al. Cho et al.	Mao et al. Turel et al.	Mao et al. Turel et al.	Turel et al.
Ethnicity	Clemons et al. Cundiff et al. Fernando et al. Geoffrion et al. Cho et al.	Cundiff et al. Cho et al.	Fernando et al.	–

### **Appendix C2** Obstetric history domain

**Table:**

Parameter	Univariate analysis assessment	Significant univariate analysis	Multivariate analysis Assessment	Significant multivariate analysis
Gravidity	Ding et al. Geoffrion et al. Mao et al. Yang et al.	–	–	–
Parity/ n. vaginal deliveries	Cheung et al. 2017 Cheung et al. 2018 Clemons et al. Ding et al. Fernando et al. Geoffrion et al. Jones et al. Ko et al. Lekskulchai et al. Manchana, 2011 Manchana et al. 2012 Mao et al. Markle et al. Mokrzycki et al. Nemeth et al. 2013 Nemeth et al. 2017 Nguyen et al. Ramsay et al. Wu et al. Yamada et al. Yang et al. Turel et al. Cho et al.	Fernando et al. Nemeth et al. 2013 Nemeth et al. 2017 Yang et al.	Fernando et al. Maito et al. Nemeth et al. 2017	Fernando et al.
Largest baby	Cheung et al. 2018 Ding et al. Geoffrion et al. Mao et al.	Geoffrion et al.	Panman et al. Geoffrion et al.	–
Assisted vaginal delivery	Geoffrion et al.	–	–	–
Tear into rectum	Geoffrion et al.	–	–	–

### **Appendix C3** (Uro) gynaecological symptoms and medications domain

**Table:**

Parameter	Univariate analysis assessment	Significant univariate analysis	Multivariate analysis assessment	Significant multivariate analysis
Urinary symptoms	Clemons et al. Ding et al. Geoffrion et al. Manchana et al. 2012 Mao et al. Markle et al. Mokrzycki et al. Nguyen et al. Ramsay et al. Wu et al. Zhu et al.	Mokrzycki et al. Nguyen et al. Wu et al.	Maito et al. Nguyen et al.	Nguyen et al.
De novo urinary incontinence	Ding et al. Ko et al. Nguyen et al.	Ko et al. Nguyen et al.	–	–
Sexually active	Cheung et al. 2017 Cheung et al. 2018 Clemons et al. Geoffrion et al. Manchana et al. 2012 Markle et al. Ramsay et al. Cho et al.	Cho et al.	–	–
Age of onset/duration symptoms	Mokrzycki et al. Yang et al.	–	–	–
Vaginal hormones	Geoffrion et al. Jones et al. Cho et al.	–	–	–
Oral hormones	Clemons et al. Geoffrion et al. Jones et al. Markle et al. Nguyen et al. Wu et al.	–	–	–
Postvoidal residual	Geoffrion et al.	–	–	–
Vaginal atrophy	Clemons et al. Mokrzycki et al. Ramsay et al.	–	–	–
Anal incontinence	–	–	Maito et al.	–
Pelvic pressure/lower backache	Nemeth et al. 2013	–	–	–
Discomfort	Ramsay et al.	–	–	–
POP necessitating manual reduction	Ramsay et al.	–	–	–

### **Appendix C4** Prior surgeries domain

**Table:**

Parameter	Univariate analysis assessment	Significant univariate analysis	Multivariate analysis Assessment	Significant multivariate analysis
Hysterectomy	Cheung et al. 2017 Cheung et al. 2018 Clemons et al. Ding et al. Fernando et al. Jones et al. Geoffrion et al. Manchana, 2011 Manchana et al. 2012 Mao et al. Markle et al. Mutone et al. Nemeth et al. 2013 Nemeth et al. 2017 Nguyen et al. Ramsay et al. Wu et al. Yamada et al. Yang et al. Turel et al. Cho et al. Hooper et al. Umachanger et al.	Fernando et al. Markle et al. Mutone et al. Nemeth et al. 2013 Nemeth et al. 2017 Ramsay et al. Cho et al. Hooper et al. Umachanger et al	Fernando et al. Maito et al. Nemeth et al. 2017 Panman et al. Turel et al.	Fernando et al. Maito et al. Turel et al.
POP surgery	Clemons et al. Ding et al. Fernando et al. Jones et al. Geoffrion et al. Mao et al. Markle et al. Mutone et al. Nemeth et al. 2013 Nemeth et al. 2017 Nguyen et al. Ramsay et al. Wu et al. Turel et al. Cho et al. Zhu et al.	Fernando et al. Mao et al. Mutone et al. Nemeth et al. 2013 Nemeth et al. 2017 Nguyen et al. Ramsay et al. Cho et al.	Fernando et al. Maito et al. Mao et al. Nemeth et al. 2017 Nguyen et al. Panman et al.	Maito et al. Nemeth et al. 2017 Nguyen et al.
Pelvic surgery	Geoffrion et al. Mokrzycki et al. Wu et al. Zhu et al. Umachanger et al.	Umachanger et al. Wu et al.	Panman et al.	–
Incontinence surgery	Geoffrion et al. Markle et al. Nguyen et al. Wu et al.	–	Maito et al.	–

### **Appendix C5** General history domain

**Table:**

Parameter	Univariate analysis assessment	Significant univariate analysis	Multivariate analysis assessment	Significant multivariate analysis
Comorbidities	Ding et al. Ko et al. Manchana et al. 2012 Yang et al.	Ko et al.	Maito et al.	–
Poor surgical candidate	Clemons et al.	–	–	–
Smoking	Ding et al. Geoffrion et al. Nguyen et al. Turel et al.	Geoffrion et al.	Geoffrion et al.	Geoffrion et al.
Family support	Ding et al. Ko et al.	Ko et al.	–	–
Constipation	Ding et al. Ramsay et al.	–	Maito et al.	–
Desire of surgery at the 1st visit	Clemons et al.	–	–	–

### **Appendix C6** Questionnaires domain

**Table:**

Parameter	Univariate analysis assessment	Significant univariate analysis	Multivariate analysis Assessment	Significant multivariate analysis
PFDI-20	Jones et al. Geoffrion et al. Yang et al	–	–	–
POPDI-6	Geoffrion et al. Yang et al.	–	–	–
UDI-6	Geoffrion et al. Yang et al.	–	–	–
CRADI-8	Geoffrion et al. Yang et al.	Yang et al.	–	–
PFIQ-7	Geoffrion et al. Yang et al.	–	–	–
POPIQ-7	Geoffrion et al. Yang et al.	–	–	–
UIQ-7	Geoffrion et al. Yang et al.	–	–	–
CRAIQ-7	Geoffrion et al. Yang et al.	–	–	–
PEQ	Geoffrion et al.	–	–	–

### **Appendix C7** POP and pelvic floor assessment domain

**Table:**

Parameter	Univariate analysis assessment	Significant univariate analysis	Multivariate analysis Assessment	Significant multivariate analysis
Compartment	Cheung et al. 2017 Cheung et al. 2018 Clemons et al. Ding et al. Fernando et al. Jones et al. Geoffrion et al. Lekskulchai et al. Mao et al. Markle et al. Mokrzycki et al. Mutone et al. Ramsay et al. Yamada et al. Turel et al. Zhu et al.	Cheung et al. 2017 Cheung et al. 2018 Lekskulchai et al. Mao et al. Mutone et al. Turel et al. Ramsay et al. Yamada et al.	Cheung et al. 2017 Cheung et al. 2018 Maito et al. Mao et al. Panman et al.	Cheung et al. 2018 Maito et al.
Stage	Cheung et al. 2017 Cheung et al. 2018 Clemons et al. Ding et al. Fernando et al. Jones et al. Geoffrion et al. Ko et al. Manchana, 2011 Manchana et al. 2012 Mao et al. Markle et al. Mokrzycki et al. Mutone et al. Nemeth et al. 2013 Nguyen et al. Ramsay et al. Yamada et al. Cho et al. Hooper et al. Zhu et al.	Cheung et al. 2017 Cheung et al. 2018 Yamada et al. Cho et al. Hooper et al.	Cheung et al. 2017 Cheung et al. 2018 Geoffrion et al. Maito et al. Panman et al.	Cheung et al. 2017 Cheung et al. 2018 Geoffrion et al.
TVL	Cheung et al. 2018 Clemons et al. Ding et al. Jones et al. Lekskulchai et al. Manchana, 2011 Mao et al. Markle et al. Mutone et al. Turel et al. Zhu et al.	Cheung et al. 2018 Clemons et al. Lekskulchai et al. Manchana 2011 Mao et al. Markle et al.	Cheung et al. 2018 Mao et al. Markle et al.	Mao et al.
Introitus width	Clemons et al. Manchana, 2011 Mao et al. Zhu et al.	Clemons et al. Manchana, 2011	–	–
GH	Cheung et al. 2018 Clemons et al. Ding et al. Jones et al. Lekskulchai et al. Manchana, 2011 Mao et al. Markle et al. Mutone et al. Turel et al.	Cheung et al. 2018	Cheung et al. 2018	Cheung et al. 2018
Pb	Cheung et al. 2018 Jones et al. Lekskulchai et al. Markle et al. Mutone et al. Turel et al. Wu et al	Turel et al. Jones et al.	Turel et al. Jones et al.	Jones et al.
GH + Pb	Turel et al.	Turel et al.	Turel et al.	Turel et al.
GH/TVL	Ding et al. Geoffrion et al. Markle et al.	Markle et al.	Geoffrion et al. Markle et al.	Geoffrion et al.
Pelvic floor strength	Geoffrion et al. Mutone et al. Turel et al.	–	Maito et al. Panman et al.	Panman et al.

TVL = total vaginal length; GH = genital hiatus; Pb = perineal body

### **Appendix C8** Pessary domain

**Table:**

Parameter	Univariate analysis assessment	Significant univariate analysis	Multivariate analysis Assessment	Significant multivariate analysis
Type	Fernando et al. Jones et al. Mutone et al. Wu et al. Cho et al.	Mutone et al. Cho et al.	Fernando et al.	–
Size	Nemeth et al. 2013	–	–	–
Self-insertion	Ding et al. Ko et al.	–	–	–
Insertion ease	Nemeth et al. 2013	Nemeth et al. 2013	–	–

### **Appendix C9** Imaging domain

**Table:**

Parameter	Univariate analysis assessment	Significant univariate analysis	Multivariate analysis Assessment	Significant multivariate analysis
TPUS	Cheung et al. 2017 Paterson et al. Turel et al.	Cheung et al. 2017 Paterson et al. Turel et al.	Cheung et al. 2017 Turel et al.	Cheung et al. 2017
MRI	Triepels et al.	Triepels et al.	–	–

TPUS = transperineal ultrasound; MRI = magnetic resonance imaging

## **Appendix E** Results of the meta-analysis including imputed data (only parameters requiring data imputation are shown)

**Table:**

**Parameter**	**OR (95% CI)**	**z-value**	**p value**	**Heterogeneity**				**Trim and fill**		**Studies included**
				**Q value**	**df (Q)**	**p value**	**I-squared**	**OR (95% CI)**	**Q value**	
**Demographics**										
**Age**	0.69 (0.58–0.82)	−4.22	**0.00**	24.26	19	0.19	21.70	0.71 (0.60–0.85)	28.35	2, 3, 4, 5, 6′, 7, 8, 10^°^, 13, 14, 15, 16, 17^°^, 18^°^, 19, 20, 21^°^, 24, 26, 27
**BMI**	1.45 (1.21–1.75)	3.93	**0.00**	10.67	10	0.38	6.28	1.29 (1.07–1.56)	17.49	2, 5′, 7, 13, 14, 15, 17′, 19, 21^°^, 24, 27
**Obstetric history**										
No. pregnancies	0.80 (0.57–1.14)	−1.24	0.22	0.84	3	0.84	0.00	–	–	5^°^, 7, 14^°^, 26
No. deliveries	0.80 (0.59–1.08)	−1.43	0.15	46.62	14	0.00	69.97	–	–	2^°^, 3, 5^°^, 6, 7, 8^°^, 10^°^, 13^°^, 18^°^, 19, 20^°^, 21^°^, 24^°^, 26, 27
No. vaginal deliveries	0.90 (0.65–1.26)	−0.60	0.55	6.05	4	0.20	33.92	–	–	2^°^, 7, 14^°^, 15, 16
Largest baby	1.53 (0.81–2.88)	1.30	0.19	10.52	4	0.03	61.97	–	–	2, 5′, 7′, 14′, 21^°^
**Questionnaires**										
**CRADI-8**	2.04 (1.37–3.04)	3.49	**0.00**	0.73	2	0.69	0.00	2.04 (1.37–3.04)	0.73	7, 21^°^, 27
**POP and pelvic floor assessment**										
**TVL**	0.44 (0.25–0.78)	−2.79	**0.01**	75.99	10	0.00	86.84	0.47 (0.27–0.83)	77.81	2, 3′, 5, 8, 10, 12′, 14^°^, 15, 17^°^, 21^°^, 24
GH	0.51 (0.91–1.62)	−0.33	0.74	90.59	10	0.00	88.96	–	–	2, 3′, 5, 8, 10^°^, 12′, 14^°^, 15, 17^°^, 21^°^, 24
Perineal body	1.24 (0.91–1.69)	1.35	0.18	10.07	5	0.07	50.36	–	–	2, 8, 10^°^, 15, 17^°^, 24
**Introitus width**	2.71 (1.61–4.58)	3.73	**0.00**	1.82	2	0.40	0.00	2.29 (1.33–3.94)	4.42	3′, 12′, 14^°^
Pelvic floor strength	0.57 (0.29–1.13)	−1.62	0.11	11.04	3	0.01	72.83	–	–	7, 17^°^, 21′, 24

**Bold** = statistically significant. *****In case of predominant multiple compartments (e.g. maximum POP stadium in the anterior and apical compartment), the patient was included in all relevant groups (e.g. predominant anterior compartment POP and predominant apical compartment POP). ^**°**^Mean and SD imputed. **‘**Only available as dichotomous variable; **nm** = not measurable (to run a publication bias procedure at least three studies must be included); **HRT** = hormone replacement therapy; **POP** = pelvic organ prolapse; **CRADI-8** = Colorectal-Anal Distress Inventory-8. The study number refers to Table 2

## Abbreviations

BMI: Body mass index
CRADI-8: Colorectal-Anal Distress Inventory-8
GH: Genital hiatus
HRT: Hormone replacement therapy
POP: Pelvic organ prolapse
SUI: Stress urinary incontinence
TPUS: Transperineal ultrasound
TVL: Total vaginal length
